# Novel Viral Communities Potentially Assisting in Carbon, Nitrogen, and Sulfur Metabolism in the Upper Slope Sediments of Mariana Trench

**DOI:** 10.1128/msystems.01358-21

**Published:** 2022-01-04

**Authors:** Jiulong Zhao, Hongmei Jing, Zengmeng Wang, Long Wang, Huahua Jian, Rui Zhang, Xiang Xiao, Feng Chen, Nianzhi Jiao, Yongyu Zhang

**Affiliations:** a Key Laboratory of Biofuels, Shandong Provincial Key Laboratory of Energy Genetics, Qingdao Institute of Bioenergy and Bioprocess Technology, Chinese Academy of Sciences, Qingdao, China; b CAS Key Laboratory for Experimental Study under Deep-Sea Extreme Conditions, Institute of Deep-Sea Science and Engineering, Chinese Academy of Sciences, Sanya, China; c University of Chinese Academy of Sciences, Beijing, China; d State Key Laboratory for Marine Environmental Science, Xiamen University, Xiamen, China; e State Key Laboratory of Microbial Metabolism, School of Life Sciences and Biotechnology, Shanghai Jiao Tong University, Shanghai, China; f University of Maryland Center for Environmental Science, Baltimore, Maryland, USA; University of Illinois at Chicago

**Keywords:** Mariana Trench sediment, metagenome, viral community, auxiliary metabolic gene

## Abstract

Viruses are ubiquitous in the oceans. Even in the deep sediments of the Mariana Trench, viruses have high productivity. However, little is known about their species composition and survival strategies in that environment. Here, we uncovered novel viral communities (3,206 viral scaffolds) in the upper slope sediments of the Mariana Trench via metagenomic analysis of 15 sediment samples. Most (99%) of the viral scaffolds lack known viral homologs, and ca. 59% of the high-quality viral genomes (total of 111 with completeness of >90%) represent novel genera, including some *Phycodnaviridae* and jumbo phages. These viruses contain various auxiliary metabolic genes (AMGs) potentially involved in organic carbon degradation, inorganic carbon fixation, denitrification, and assimilatory sulfate reduction, etc. This study provides novel insight into the almost unknown benthic viral communities in the Mariana Trench.

**IMPORTANCE** The Mariana Trench harbors a substantial number of infective viral particles. However, very little is known about the identity, survival strategy, and potential functions of viruses in the trench sediments. Here, through metagenomic analysis, unusual benthic viral communities with high diversity and novelty were discovered. Among them, 59% of the viruses with a genome completeness of >90% represent novel genera. Various auxiliary metabolic genes carried by these viruses reflect the potential adaptive characteristics of viruses in this extreme environment and the biogeochemical cycles that they may participate in. This study gives us a deeper understanding of the peculiarities of viral communities in deep-sea/hadal sediments.

## INTRODUCTION

The hadal zone, or hadopelagic zone, is the region of the ocean deeper than 6,000 m, lying within oceanic trenches (1). Hadal trenches account for the deepest 45% of the oceanic depth range (1). These places are characterized by their harsh environmental conditions, with high hydrostatic pressures (up to 1.1 tonnes per cm^2^) and near-freezing low temperatures (2, 3). Meanwhile, hadal trenches, like traps, capture various substances and contain diverse novel prokaryotes with special metabolic characteristics (4–6). For example, numerous microorganisms that can consume refractory organic matter (e.g., *Oleibacter*, *Alcanivorax*, and *Chloroflexi*) or have chemoautotrophic functions (e.g., *Thaumarchaeota*, *Nitrospirae*, and *Nitrospinae*) become dominant groups and form unique clades in hadal environments (2, 5, 7, 8). As well as being affected by the shaping effect of environmental factors, microbial communities are also regulated by viral infections (9, 10). This is especially so in environments such as deep-sea sediments where other microbial predators (e.g., zooplankton) are somewhat restricted (11, 12). Here, the regulatory roles of viruses are far more significant. For example, the fraction of prokaryote mortality due to viral lysis increases with water depth, with reported proportions of ca. 16% in coastal sediments, ca. 64% in mesopelagic sediments, and ca. 89% in deep-sea sediments at depths of >1,000 m (12). Most recently, it was reported that viral infection is also an important factor determining microbial mortality in the hadal sediments (13). Moreover, many viruses contain auxiliary metabolic genes (AMGs), and they can remodel the metabolism of host cells during infection. Indeed, many bacteria in the ocean are phage-infected hosts, termed virocells, and they are in a metabolically distinct state (14). However, so far, little is known about these benthic viruses in terms of their identity, their survival strategy, and whether they contain novel auxiliary metabolic genes in this extreme environment.

**FIG 1:**
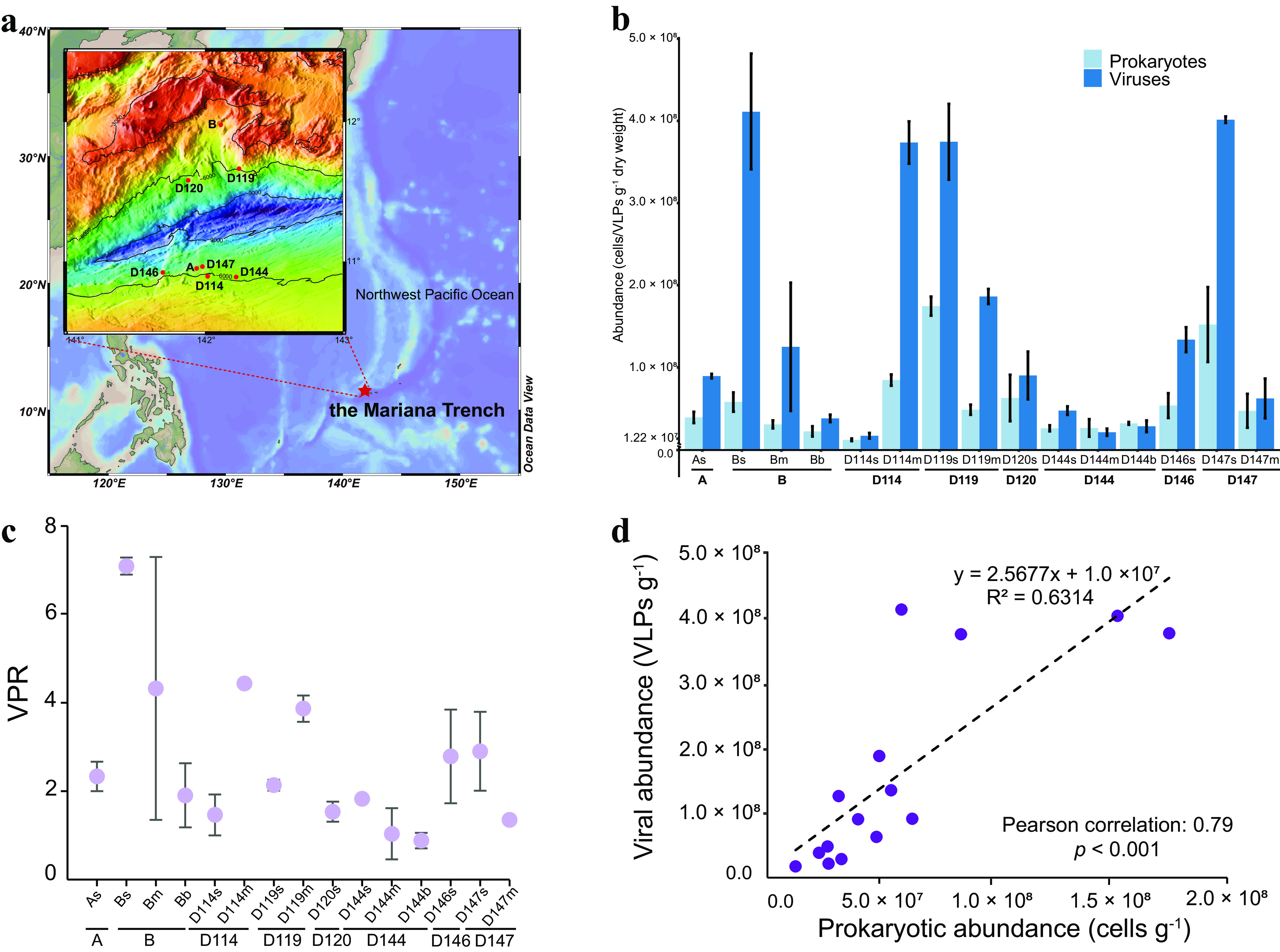
Sampling locations and abundance profiles of the MTS viruses and prokaryotes. (a) Satellite image of the sampling location (red star) in the Mariana Trench of the Pacific Ocean and sampling sites and depths. (b) Enumerated abundances of viruses and prokaryotes in each sample. (c) Virus-to-prokaryote ratio (VPR) of each sample, calculated as the viral abundance divided by the prokaryotic abundance. For both panels b and c, “A, B, D114, D119, D120, D144, D146, and D147” represent different sampling sites, and the following “s,” “m,” and “b” represent the sediment samples collected from the pushcore of 0-6 cm (surface part), 6-12 cm (middle part), and 12-18 cm (bottom part), respectively (see Materials and Methods). (d) Relationship between prokaryotic and viral abundances in MTS (*P < *0.001 by linear regression). In panels b and c, the data are shown as means ± standard deviations.

The Mariana Trench is located in the Northwest Pacific Ocean (Fig. 1a) (15). Despite the extreme environmental conditions that exist in the Mariana Trench, there is still a high prokaryotic biomass (approximately 2.01 μg C g^−1^) and activity in the sediments of this region (10). Concurrently, viruses, which are major predators of microbial communities, also have high abundances (approximately 10^7^ virus-like particles [VLPs] per g or cm^3^ sediments), with viral production of ca. 10^6^ viruses h^−1^ g^−1^ in the sediments of this trench (9, 10, 13, 16–18). Multiple factors can affect the structure and function of virus communities. These include physicochemical conditions (such as nutrients, light, temperature, and salinity) and host community characteristics (19, 20). In view of the extreme environmental conditions and the uniqueness of the host microbial communities inhabiting the Mariana Trench sediments (MTSs) (5–7, 21), we speculated that the virus communities there might have some unusual characteristics, which will enhance our understanding of marine viruses.

During two cruises in 2016 and 2017, a total of 15 surface sediment samples were collected from the upper slopes of the Mariana Trench at depths of 5,481 to 6,707 m (see Table S1 at https://doi.org/10.6084/m9.figshare.c.5703367.v6). Here, novel viral communities were identified, and 111 high-quality benthic viral genomes were obtained. Of particular interest was that numerous novel viruses form independent branches throughout the phylogenetic trees. In addition, many auxiliary metabolic genes that assist in organic carbon degradation, inorganic carbon fixation, denitrification, and assimilatory sulfate reduction were discovered in the benthic viral metagenomes of the Mariana Trench.

## RESULTS

### Environmental parameters and overview of the benthic viromes.

Fifteen sediment samples were collected from eight different locations at various depths, mainly in the upper area of the north and south slopes of the Mariana Trench (5,481 to 6,707 m) (Fig. 1; see also Table S1 at https://doi.org/10.6084/m9.figshare.c.5703367.v6). As for the environmental parameters of the sediments (11 samples available) in this study, no significant variation was observed among different sampling sites or sediment depths, except for the D147 site, with significantly lower ammonia nitrogen (NH_4_-N) levels (*P < *0.05 by a permutation test) (see Fig. S1a in the supplemental material). Compared with the adjacent abyssal plain sediments (mainly in the Northwestern Pacific), the upper slope sediments of the Mariana Trench of this study are a relatively oligotrophic environment with significantly lower levels of nutrients (such as total carbon [TC] and total nitrogen [TN]) (*P < *0.001) (Fig. S1b and c; see also Table S2 at https://doi.org/10.6084/m9.figshare.c.5703367.v6). Of all the sediment samples, the δ^13^C values ranged from −26.41‰ to −22.81‰ (average, −24.14‰), and the δ^15^N values ranged from 4.05‰ to 7.67‰ (average, 6.29‰), both suggesting that the organic matter therein was mainly derived from phytoplankton (22).

10.1128/msystems.01358-21.2 FIG S1 Physicochemical properties of the Mariana Trench slope sediments. (a) Bubble charts showing the variations in total phosphorus (TP), total carbon (TC), total nitrogen (TN), nitrate-nitrogen (NO_3_-N), and ammonia nitrogen (NH_4_-N) in the different samples. The *y* axis represents 11 different samples, belonging to five sediment cores, in different colors. The *x* axis and the size of each bubble indicate the content of each parameter. (b to d) Box-and-jitter plots showing comparisons of TN (b) and TC (dark blue stars) or total organic carbon (TOC) (light blue stars) (c) and C/N ratios between the different deep-sea sediment locations (d). The *x* axis represents the different sampling locations, including the Mariana Trench slopes (this study) and the surrounding deep-sea seafloor (previously published data [see references 6 to 9 in Text S1] and data retrieved from the IODP database). The upper and lower lines of each box correspond to the 25th and 75th percentiles, respectively. Significance letters are shown over the boxes. ***, *P < *0.001; **, *P < *0.01; *, *P < *0.05. n, sample size. Download FIG S1, TIF file, 1.8 MB. Copyright © 2022 Zhao et al. 2022 Zhao et al. https://creativecommons.org/licenses/by/4.0/ This content is distributed under the terms of the Creative Commons Attribution 4.0 International license.

The mean abundances of viruses and prokaryotes in the sediments ranged from 1.72 × 10^7^ to 4.11 × 10^8^ virus-like particles (VLPs)/g (dry weight) and 1.22 × 10^7^ to 1.75 × 10^8^ cells/g (dry weight), respectively (Fig. 1b). The abundance of benthic viruses is comparable to that in the bottom sediments of the Mariana Trench (10,325 m and 10,901 m) and the adjacent abyssal plain (10, 16) but relatively lower than those in many bathypelagic or abyssopelagic sediments, which are up to 10^9^ to 10^10^ VLPs/g (9, 23–25). However, the virus-to-prokaryote ratio (VPR) values (from 0.88 to 7.09) in the Mariana Trench sediments (Fig. 1c) were similar to those in bathypelagic or abyssopelagic sediments, as the prokaryotic abundance in the Mariana Trench sediments is also relatively low (12, 23, 25–27). Although the abundances of viruses (*F* = 17.87; *P < *0.001) and prokaryotes (*F* = 9.194; *P < *0.001) varied significantly at different sampling sites, there was a significant positive correlation between virus and prokaryote (Pearson correlation coefficient = 0.79; *P < *0.001) (Fig. 1d).

**FIG 2:**
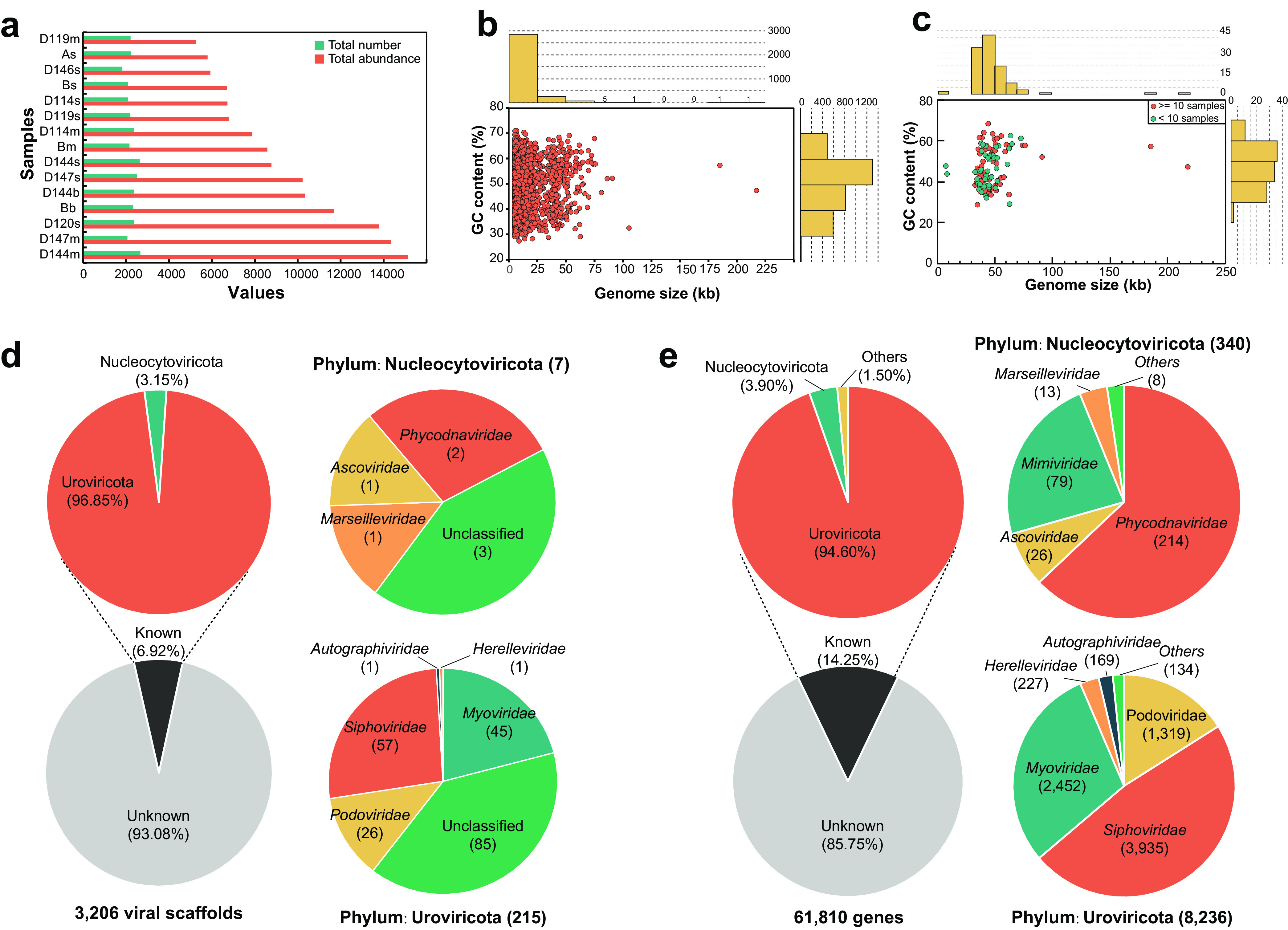
Profiles of MTS-VSs and their taxonomic assignments. (a) Total number (green) and abundance (red) of VSs (*x* axis) in each sediment sample (*y* axis). The samples are shown by increasing richness. (b) Distribution of genome size and GC content (percent) of 3,206 viral scaffolds. Each circle represents one viral scaffold. (c) Distribution of genome size and GC content (percent) of 111 high-quality viral scaffolds. Each circle represents one circular viral scaffold. The red circles represent the viruses that exist in 10 or more samples, whereas the others are represented by green circles. (d) Taxonomic assignments of all viral scaffolds. (e) Taxonomic assignments of all predicted viral protein-coding sequences.

Through metagenomic sequencing and bioinformatic analysis of 15 sediment metagenomes of the Mariana Trench, a data set named MTS-VSs (Mariana Trench sediment viral scaffolds) was generated. This data set consists of 3,206 viral scaffolds (each scaffold is ≥5 kb, added up to ca. 40 Mbp of DNA sequences) and 61,810 open reading frames (ORFs). With regard to the viral scaffolds, in this data set, 1,012 were >10 kb, 3 were >100 kb, and 1 (the longest) was 217,265 bp (see Table S1 at https://doi.org/10.6084/m9.figshare.c.5703367.v6). Each scaffold represents a viral cluster based on shared gene content at the subfamily/genus level. The number of these viral scaffolds in every sediment metagenome ranged from 1,807 to 2,666 (Fig. 2a; see also Table S1 at https://doi.org/10.6084/m9.figshare.c.5703367.v6). The GC content of each viral scaffold ranged from 27.40% to 70.98% (mean, 50.07%) (Fig. 2b). In the MTS-VSs data set, 111 viral scaffolds were predicted as high-quality viral genomes (>90% completeness), 82 of which were complete viral genomes (100% completeness and zero contamination) (see Table S3 at https://doi.org/10.6084/m9.figshare.c.5703367.v6). Among these high-quality viral genomes, most (103 out of 111) were 30 to 70 kb in length, and the longest and shortest ones were ∼217 kb and ∼7 kb in length, respectively (Fig. 2c).

### Distribution of viral and prokaryotic communities in the trench sediments.

The host prokaryotic community structures in the trench slope sediments were also analyzed based on their metagenomic sequences. Among them, *Proteobacteria* (mainly *Gammaproteobacteria* and *Alphaproteobacteria*), *Thaumarchaeota*, *Chloroflexi*, *Planctomycetes*, *Acidobacteria*, *Gemmatimonadetes*, *Actinobacteria*, *Bacteroidetes*, *Cyanobacteria*, and *Nitrospirae* were the most dominant groups at the phylum level (Fig. S2a). In addition, at the genus level, *Woeseia*, *Gemmatimonas*, *Nitrosopumilus*, “*Candidatus* Entotheonella,” *Phycisphaera*, Pseudomonas, *Bradyrhizobium*, *Nitrospina*, *Tistlia*, and “*Candidatus* Nitrosoarchaeum” were the most abundant prokaryotes (Fig. S2b).

10.1128/msystems.01358-21.3 FIG S2 Relative abundances of prokaryotic sequences in the metagenomes of the Mariana Trench sediments. (a) Taxonomic composition of the sequences at the phylum level, with ranks ordered from top to bottom by their increasing proportions. Groups with a <0.3% abundance and unclassified groups are labeled “Other.” The red and yellow backgrounds represent archaea and bacteria, respectively. (b) Heat map showing the taxonomic composition of sequences at the genus level, with ranks ordered from top to bottom by their increasing proportions. Groups with a >0.1% relative abundance (after log_10_ transformation) are shown. The phylum (or class for *Proteobacteria*) information is in red. Download FIG S2, TIF file, 2.5 MB. Copyright © 2022 Zhao et al. 2022 Zhao et al. https://creativecommons.org/licenses/by/4.0/ This content is distributed under the terms of the Creative Commons Attribution 4.0 International license.

The virus communities (Fig. S3a) displayed variation patterns in alpha diversity indexes (Chao1 and Simpson) similar to those of the prokaryotes (Fig. S3b). At some sites (such as sites B, D114, D119, and D144), the viruses exhibited lower community diversity and higher richness with an increase in sediment depth (Fig. S3a). No significant spatial patterns in the prokaryote or virus community structure were observed by principal-coordinate analysis (PCoA) and nonmetric multidimensional scaling (NMDS) analysis despite that the *P* values by ADONIS (permutational multivariate analysis of variance using distance matrices) and ANOSIM (analysis of similarity) were <0.05 (Fig. S3c to f). In addition, distance-based redundancy analysis (db-RDA) showed that the benthic viral communities were most affected by NO_3_-N (explains, 31.01%; *P < *0.05) and latitude (explains, 22.42%; *P < *0.05) (Fig. S3g).

10.1128/msystems.01358-21.4 FIG S3 Alpha and beta diversities of the viral and prokaryotic communities. (a and b) Alpha diversity indexes (Chao1 and Simpson) of the communities of viruses (black dots) (a) and prokaryotes (gray dots) (b). The *x* axis represents samples collected from different sites and locations (i.e., north or south slope). (c and d) Principal-coordinate analysis (PCoA) of virus (c) and prokaryote (d) communities among the different sampling sites. (e and f) Nonmetric multidimensional scaling (NMDS) of the virus (e) and prokaryote (f) communities among the different sampling sites. (g and h) Distance-based redundancy analysis (db-RDA) of the relationship between environmental factors and the communities of viruses (g) and prokaryotes (h). Arrows show significant environmental variables after forward selection. Asterisks indicate the significant variables (*P < *0.05). In panels c to h, the different sampling sites are represented by different circles, and s, m, and b are “surface,” “middle,” and “bottom,” respectively. Download FIG S3, TIF file, 2.3 MB. Copyright © 2022 Zhao et al. 2022 Zhao et al. https://creativecommons.org/licenses/by/4.0/ This content is distributed under the terms of the Creative Commons Attribution 4.0 International license.

### Novelty of the genetic profile of MTS viruses.

The vast majority of the viruses in the upper slope sediments of the Mariana Trench were previously unknown. Specifically, DNA sequence comparisons showed that 99.28% (3,183 out of 3,206) of the viral scaffolds were novel as they did not have any homolog in the Integrated Microbial Genome/Virus (IMG/VR) 3.0 database (see Table S4 at https://doi.org/10.6084/m9.figshare.c.5703367.v6). Even among the remaining 23 viral scaffolds with homologs, 22 were uncultured marine or sediment viruses (see Table S4 at https://doi.org/10.6084/m9.figshare.c.5703367.v6). In addition, 93.08% of all the viral scaffolds (i.e., 2,984 out of 3,206) (see Table S3 at https://doi.org/10.6084/m9.figshare.c.5703367.v6) could not be determined with a clear taxonomic identification. Among others, 7 viral scaffolds were from the nucleocytoplasmic large DNA viruses (NCLDVs), containing members of the *Ascoviridae*, *Phycodnaviridae*, and *Marseilleviridae* affiliated with the *Nucleocytoviricota* phylum (28, 29) (Fig. 2d; see also Table S3 at https://doi.org/10.6084/m9.figshare.c.5703367.v6); 215 viral scaffolds were from members of the *Uroviricota* phylum (*Caudovirales* order) (Fig. 2d).

**FIG 3:**
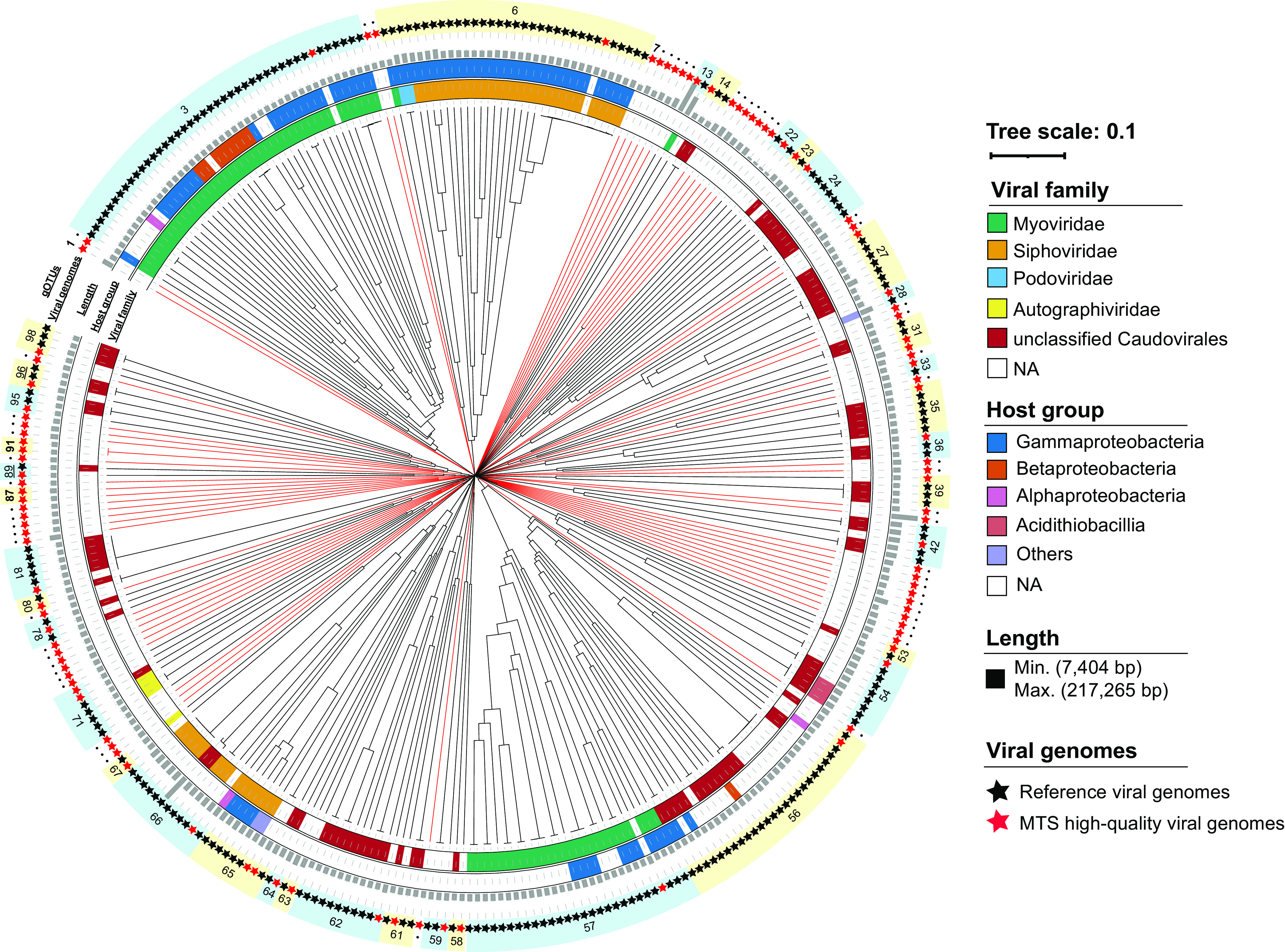
Proteomic tree of MTS viruses and reference viruses. The tree represents proteome-wide similarity relationships among 103 MTS high-quality viral genomes and 230 reference viral genomes. The dendrogram is midpoint rooted. From inside to outside, the rings outside the tree represent the viral family, host group, genome length, MTS (red) or reference (black) viral genomes, and gOTUs, respectively. The serial numbers represent gOTUs, and those containing only MTS viruses are in boldface type. The red branches represent the novel gOTUs composed of only MTS viruses. NA, not applicable.

Based on the genomic similarity (*S_G_* score) matrix, a proteomic tree containing 103 high-quality viral scaffolds and 230 reference viral genomes was constructed, which shows a long evolutionary distance between the branches of the MTS viral genomes and the reference viral genomes (Fig. 3). Using a threshold of an *S_G_* of >0.15, the best threshold for genus-level clustering of the viral genomic operational taxonomic units (gOTUs) (30), the 103 high-quality viral scaffolds were assigned to 98 different viral gOTUs. Among these, 62 gOTUs could not be clustered with any other reference viral genome and were therefore determined to be novel viral genera (Fig. 3). In addition, the proteomic tree of eukaryotic viruses (see Fig. S11 at https://doi.org/10.6084/m9.figshare.c.5703367.v6) showed that the other 8 high-quality viral scaffolds were assigned to 7 different viral gOTUs. However, each viral gOTU contained reference genomes of known viruses infecting eukaryotic algae and chordates.

Further analysis at the gene level revealed that only 8,807 (14.25%) and 27,706 (44.82%) of the viral genes (total of 61,810) in the MTS-VSs data set had homologs in the RefSeq virus protein database (Fig. 2e) and the IMG/VR v3.0 (proteins) database (see Table S5 at https://doi.org/10.6084/m9.figshare.c.5703367.v6), respectively. Among the homologs in the RefSeq virus protein database, 94.60% were from the *Caudovirales* order (dominated by *Siphoviridae*, *Myoviridae*, and *Podoviridae*) of the *Uroviricota* phylum, and 3.90% were from *Nucleocytoviricota* (Fig. 2e). Phylogenetic analysis based on the phage terminase large-subunit domain gene (*terL*) also showed that the MTS viruses formed numerous independent and relatively long branches throughout the phylogenetic tree (Fig. S4), indicating their high diversity and novelty.

10.1128/msystems.01358-21.5 FIG S4 Maximum likelihood phylogenetic tree of the *Caudovirales* terminase large-subunit domain genes (*terL* genes) (1,000 iterations; LG+R10 model). The phylogenetic tree was constructed from amino acid sequences of 73 MTS viral TerL and 2,534 reference TerL sequences from the IMG/VR 3.0 database (protein). Bootstrap values of >50% are indicated with a black dot. The MTS viral TerL branches are in red, and the others in black are affiliated with the reference TerL sequence. Download FIG S4, TIF file, 2.0 MB. Copyright © 2022 Zhao et al. 2022 Zhao et al. https://creativecommons.org/licenses/by/4.0/ This content is distributed under the terms of the Creative Commons Attribution 4.0 International license.

Meanwhile, 374 viral scaffolds were predicted to be potential proviruses (see Table S3 at https://doi.org/10.6084/m9.figshare.c.5703367.v6), and 181 different ORFs were annotated as virus-encoded lysogenic infection markers (i.e., integrase, invertase, serine recombinase, and CI/Cro repressor) (see Table S6 at https://doi.org/10.6084/m9.figshare.c.5703367.v6).

### Functional content and AMGs of the MTS viruses.

The predicted ORFs of the MTS viral scaffolds were annotated by searching against the clusters of orthologous groups (COG) in the eggNOG database. Only 16.69% of the viral ORFs (10,315 out of 61,810) were assigned to certain COG classes (see Table S5 at https://doi.org/10.6084/m9.figshare.c.5703367.v6), among which as many as 42.39% (4,373 out of 10,315) were also found to belong to the “function unknown” class (Fig. S5a and b; see also Table S5 at https://doi.org/10.6084/m9.figshare.c.5703367.v6). The ORFs with annotated functions were mainly assigned to “replication, recombination, and repair”; “transcription”; “translation, ribosomal structure, and biogenesis”; and “cell wall/membrane/envelope biogenesis,” containing viral structural proteins, all of which are known to be critical for virus reproduction and morphogenesis (Fig. S5a and b).

10.1128/msystems.01358-21.6 FIG S5 Function annotation of the MTS viromes. (a) Box plot showing the total absolute abundance of each COG functional class, ordered by hit frequency. The upper and lower lines of each box correspond to the 25th and 75th percentiles, respectively, and outliers are displayed as points. (b) Rose chart showing the number of unique genes classified by COG function. Download FIG S5, TIF file, 1.2 MB. Copyright © 2022 Zhao et al. 2022 Zhao et al. https://creativecommons.org/licenses/by/4.0/ This content is distributed under the terms of the Creative Commons Attribution 4.0 International license.

Many viral auxiliary metabolic genes (AMGs) were also discovered by viral protein annotation using VIBRANT and DRAM-v software with manual curation. These AMGs were involved in a variety of metabolic pathways, such as carbohydrate metabolism, inorganic carbon fixation, nitrogen metabolism, sulfur metabolism, and cofactor biosynthesis (see Table S8 at https://doi.org/10.6084/m9.figshare.c.5703367.v6). *In silico*, 22 AMGs had strongly supported (>98% confidence) structure predictions as enzymes participating in carbon, nitrogen, sulfur metabolism, and cofactor biosynthesis, and ca. 68% of them contained the conserved functional domains (see Table S9 at https://doi.org/10.6084/m9.figshare.c.5703367.v6).

**FIG 4:**
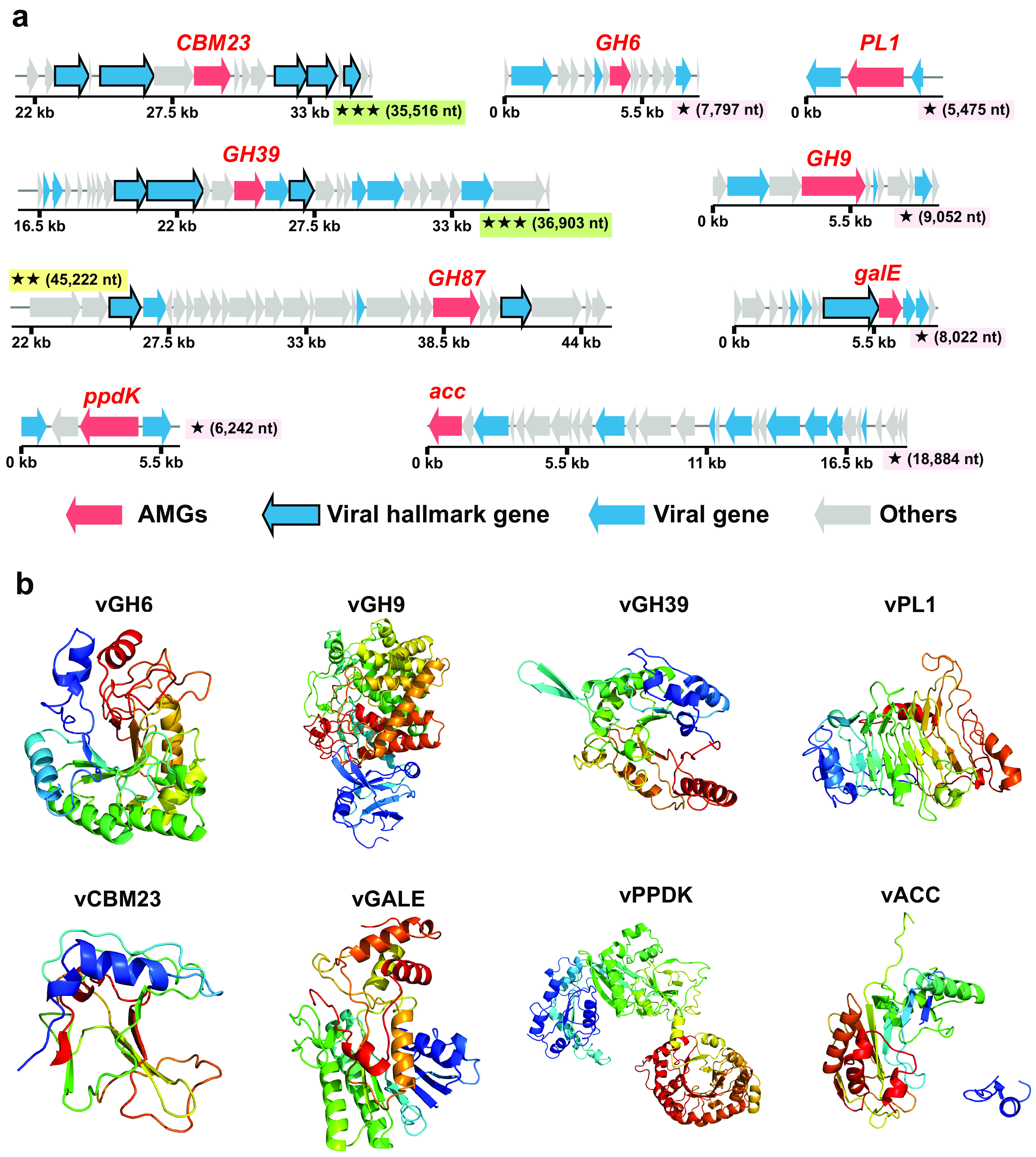
Genomic context and predicted protein structures of AMGs involved in carbon metabolism. (a) Genomic maps of AMG-containing viral scaffolds. The genome quality (green rectangles with three stars for high quality, yellow rectangles with two stars for medium quality, and pink rectangles with one star for low quality or not determined) and length are shown near the maps. AMGs are in red, virus-like genes are in blue (viral hallmark genes are framed), and non-virus-like or uncharacterized genes are in gray. Detailed annotations can be found in Table S10 at https://doi.org/10.6084/m9.figshare.c.5703367.v6. (b) Tertiary structures of viral proteins expressed by viral AMGs involved in carbon metabolism.

Specifically, half (11 out of 22) of these high-confidence viral AMGs were involved in carbon metabolism. Among them, seven viral AMGs were involved in organic carbon degradation, including the genes of glycoside hydrolases (GH6, GH9, GH39, and GH87), polysaccharide lyase (PL1), and a carbohydrate-binding module (CBM23) (Fig. 4a; see also Table S9 at https://doi.org/10.6084/m9.figshare.c.5703367.v6). Viral GH6, GH9, and GH39 family proteins were homologous to cellulases; the viral PL1 protein was homologous to pectate lyase. These viral carbohydrate metabolism enzymes might contribute to the degradation of polysaccharides in deep-sea sediments. Besides, there was a viral AMG encoding UDP-glucose 4-epimerase involved in galactose metabolism (Fig. 4; see also Table S9 at https://doi.org/10.6084/m9.figshare.c.5703367.v6), likely contributing to energy production. Surprisingly, we also discovered two viral AMGs encoding pyruvate phosphate dikinase (PPDK) and one viral AMG encoding acetyl-CoA carboxylase (ACC), which potentially participate in inorganic carbon fixation (Fig. 4; see also Table S9 at https://doi.org/10.6084/m9.figshare.c.5703367.v6). PPDK participates in the reductive tricarboxylic acid (rTCA) and dicarboxylate/4-hydroxybutyrate (DC/4-HB) cycles, and ACC participates in the 3-hydroxypropionate (3-HP) or 3-HP/4-hydroxybutyrate (4-HB) cycles (31), which are the main pathways for dark carbon fixation by deep-sea microbes (31).

**FIG 5:**
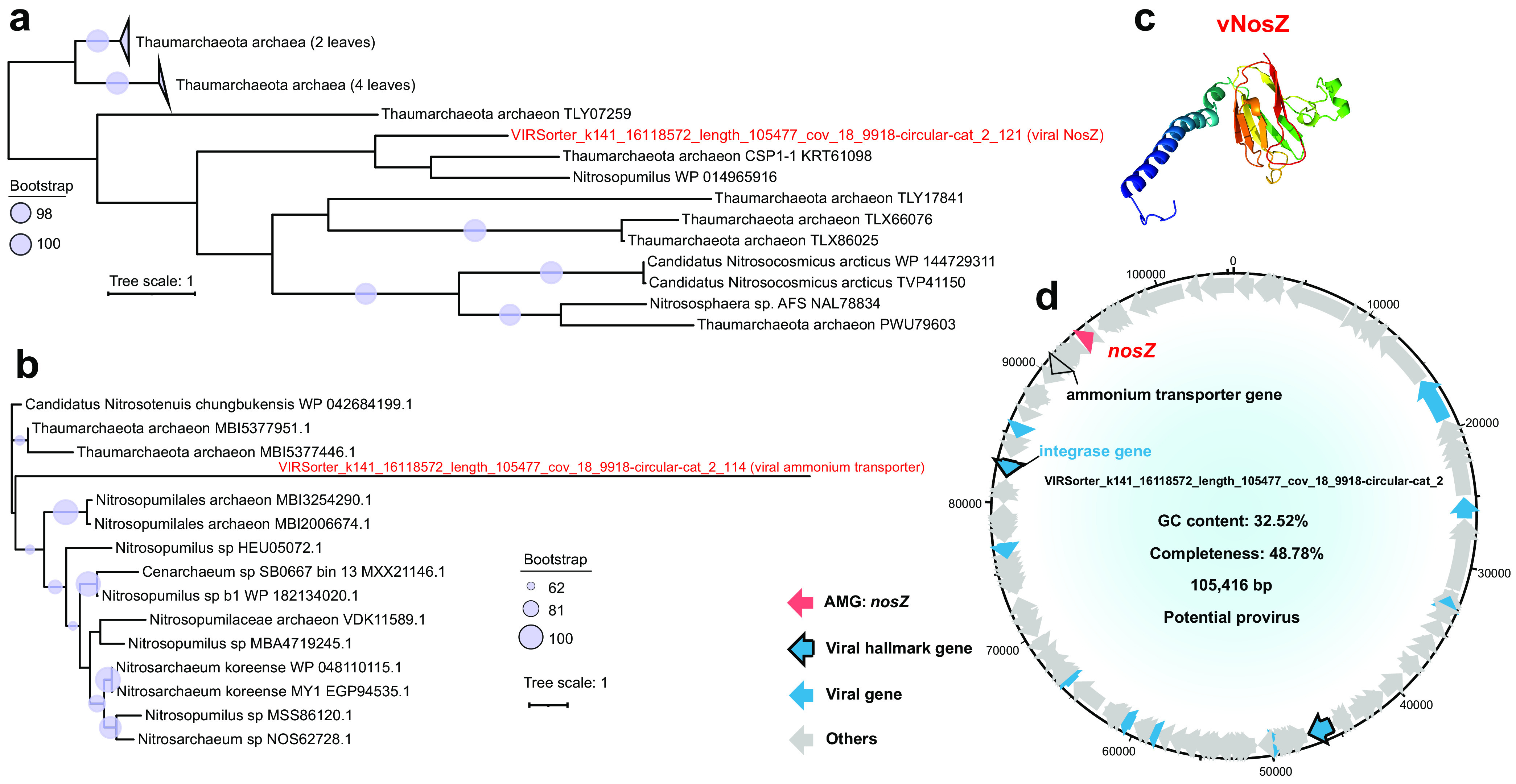
Genomic context, predicted protein structure, and phylogeny of the viral *nosZ* gene. (a and b) Maximum likelihood trees (from an amino acid alignment) of the viral *nosZ* gene, including 1 MTS viral NosZ sequence and 16 reference sequences (a), and a viral ammonium transporter gene, including 1 MTS viral sequence and 14 reference sequences (b). The proportional circles represent internal nodes and bootstraps. For panel a, those clades with an average branch length distance to leaves of <0.4 are collapsed. (c) Tertiary structure of the viral NosZ protein. (d) Genomic map of *nosZ*-containing viral scaffolds. Viral *nosZ* genes are in red, virus-like genes are in blue (viral hallmark genes are framed), and non-virus-like or uncharacterized genes are in gray. The ammonium transporter gene is framed and in gray.

Moreover, there is a viral AMG (*nosZ* gene) assisting in nitrogen metabolism (see Table S9 at https://doi.org/10.6084/m9.figshare.c.5703367.v6). The *nosZ* gene encodes nitrous oxide reductase (NosZ) catalyzing the final step of denitrification, i.e., the reduction of nitrous oxide to dinitrogen (32). Notably, in the *nosZ*-containing circular viral genome, 17 out of 25 annotated proteins (total of 130 viral proteins) were homologous to their counterparts from the archeal phylum *Thaumarchaeota*, including viral NosZ and an ammonium transporter (Fig. 5a, b, and d; see also Table S5 at https://doi.org/10.6084/m9.figshare.c.5703367.v6).

**FIG 6:**
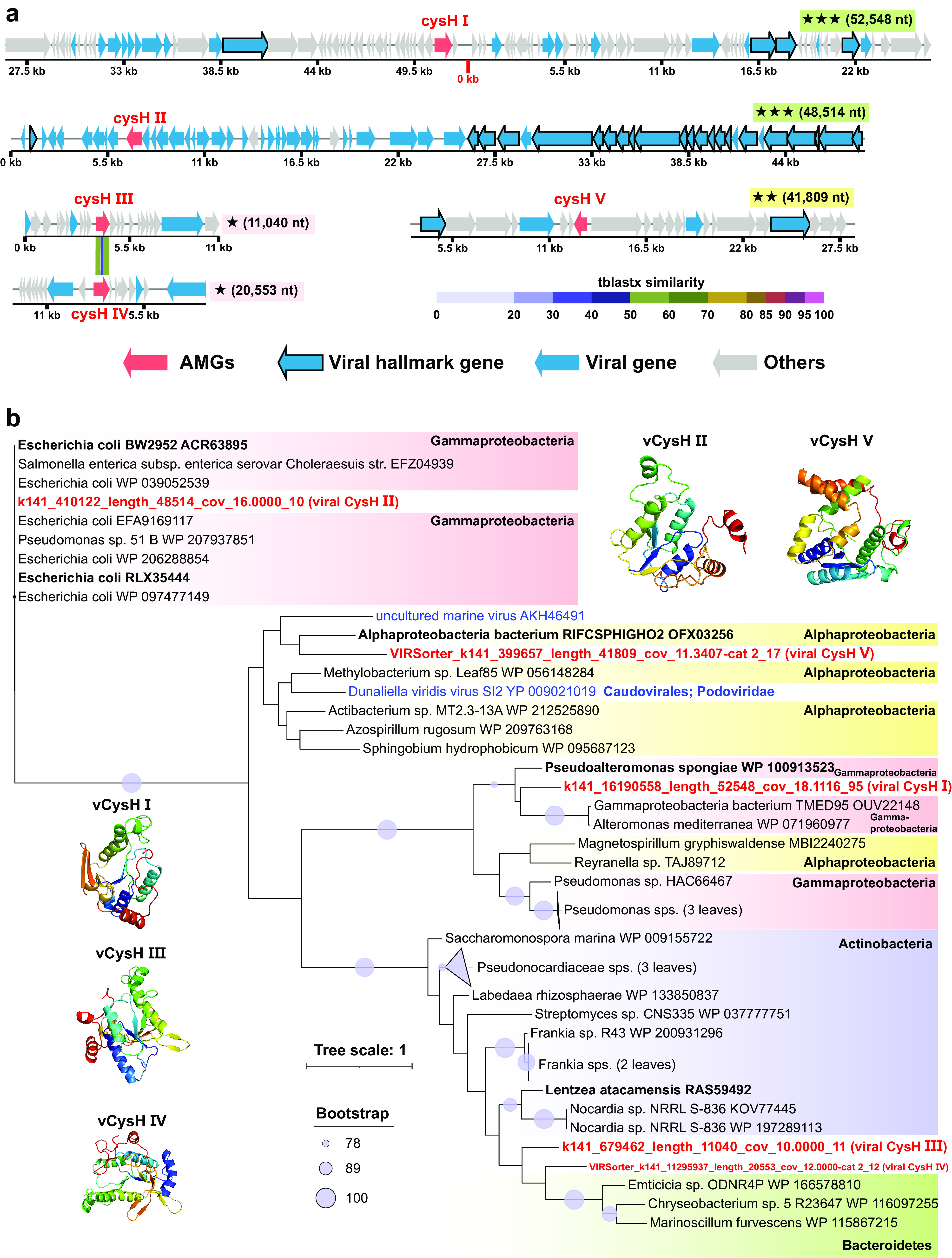
Genomic context, predicted protein structure, and phylogeny of the viral *cysH* gene. (a) Genomic maps of *cysH*-containing viral scaffolds. The genome quality (green rectangles with three stars for high quality, yellow rectangles with two stars for medium quality, and pink rectangles with one star for low quality or not determined) and length are shown near the maps. Viral *cysH* genes are in red, virus-like genes are in blue (viral hallmark genes are framed), and non-virus-like or uncharacterized genes are in gray. Between two similar *cysH* genes (III and IV), tBLASTx alignment is represented by colored lines, and their percent identity is represented by the color scale. Detailed annotations can be found in Table S10 at https://doi.org/10.6084/m9.figshare.c.5703367.v6. (b) Maximum likelihood tree (from an amino acid alignment) of the viral *cysH* gene, including 5 MTS viral *cysH* sequences (in red) and 39 reference sequences (two viral sequences are in blue), and tertiary structures of these viral CysH proteins. The most closely related reference sequences are highlighted in boldface type. The proportional circles represent internal nodes and bootstraps, and the clades where the average branch length distance to leaves is <0.4 are collapsed.

Meanwhile, six viral AMGs involved in sulfur metabolism were also identified (see Table S9 at https://doi.org/10.6084/m9.figshare.c.5703367.v6). Among them, five AMGs (*cysH* I to V) encoding phosphoadenosine phosphosulfate reductases (CysH) (Fig. 6) participate in assimilatory sulfate reduction (33). The remaining one was a viral *cysK* gene encoding cysteine synthase (CysK), which catalyzes the synthesis of l-cysteine from sulfide as well as the reverse reaction.

The viral AMGs usually come from the host through horizontal gene transfer (34). In order to predict the potential source of these viral AMGs, we conducted a phylogenetic analysis of these AMGs and reference host genes (35–37). The AMGs involved in the degradation of organic carbon (mainly cellulose and pectin) were likely transferred horizontally from *Gemmatimonadetes*, *Betaproteobacteria*, and *Gammaproteobacteria* (Fig. S6). The viral PPDK gene potentially involved in carbon fixation might be transferred from *Thaumarchaeota* and *Alphaproteobacteria* (Fig. S7). The viral *nosZ* genes involved in nitrogen metabolism might be transferred from *Thaumarchaeota* (Fig. 5a and b). As for the AMGs involved in sulfur metabolism, viral *cysH* genes (I to V) were potentially transferred from *Gammaproteobacteria*, *Actinobacteria*, and *Alphaproteobacteria* (Fig. 6b), while the viral *cysK* gene might be transferred from *Thaumarchaeota* (Fig. S8c).

10.1128/msystems.01358-21.7 FIG S6 Phylogenies of carbohydrate metabolism enzymes (carbohydrate-active enzymes [CAZymes]) encoded by the MTS viruses. The maximum likelihood trees (from an amino acid alignment) include four MTS viral CAZyme sequences and reference sequences. The proportional circles represent internal nodes and bootstraps, and the clades where the average branch length distance to leaves is <0.4 are collapsed. The MTS viral CAZyme sequences are in red, and the taxonomic information for each branch is shown in yellow. The most closely related reference sequences are highlighted in boldface type. Download FIG S6, TIF file, 2.1 MB. Copyright © 2022 Zhao et al. 2022 Zhao et al. https://creativecommons.org/licenses/by/4.0/ This content is distributed under the terms of the Creative Commons Attribution 4.0 International license.

10.1128/msystems.01358-21.8 FIG S7 Phylogenies of PPDK encoded by the MTS viruses. A maximum likelihood tree (from an amino acid alignment) of pyruvate phosphate dikinases is shown. The proportional circles represent internal nodes and bootstraps. Those clades where the average branch length distance to leaves is <0.4 are collapsed. The MTS viral PPDK enzyme sequences are in red, and the most closely related reference sequences are highlighted in boldface type. Download FIG S7, TIF file, 0.7 MB. Copyright © 2022 Zhao et al. 2022 Zhao et al. https://creativecommons.org/licenses/by/4.0/ This content is distributed under the terms of the Creative Commons Attribution 4.0 International license.

10.1128/msystems.01358-21.9 FIG S8 Genomic context, predicted protein structure, and phylogeny of the viral *cysK* gene. (a) Genomic map of the *cysK*-containing viral scaffold. The genome quality (pink rectangles with one star for low quality or not determined) and length are shown near the maps. The viral *cysK* gene is in red, virus-like genes are in blue, and non-virus-like or uncharacterized genes are in gray. (b) Tertiary structure of the viral CysK protein. (c) Maximum likelihood tree (from an amino acid alignment) of the viral *cysK* gene, including 1 MTS viral CysK sequence (in red) and 27 reference sequences. The most closely related reference sequences are highlighted in boldface type. The proportional circles represent internal nodes and bootstraps. Download FIG S8, TIF file, 1.8 MB. Copyright © 2022 Zhao et al. 2022 Zhao et al. https://creativecommons.org/licenses/by/4.0/ This content is distributed under the terms of the Creative Commons Attribution 4.0 International license.

As for the viral AMGs involved in cofactor biosynthesis (see Table S9 at https://doi.org/10.6084/m9.figshare.c.5703367.v6), four viral *cobS* genes encoding cobaltochelatases (CobS) participate in cobalamin (vitamin B_12_) biosynthesis, one of which was present in the longest complete viral genome (∼217 kb), i.e., the genome of a jumbo phage. In the genome of this jumbo phage, there were 263 functionally unknown genes, 4 DNA/RNA polymerase genes, 42 tRNA genes, and 28 structural protein genes (Fig. S9).

10.1128/msystems.01358-21.10 FIG S9 Genomic context and predicted protein structure of the viral *cobS* gene. A genomic map of the longest complete viral genome containing the *cobS* gene and the tertiary structure of this viral CobS protein are shown. The viral *cobS* gene is in red, virus-like genes are in blue (viral hallmark genes are framed), and non-virus-like or uncharacterized genes are in gray. The predicted tRNA genes are shown in the innermost track. Download FIG S9, TIF file, 1.8 MB. Copyright © 2022 Zhao et al. 2022 Zhao et al. https://creativecommons.org/licenses/by/4.0/ This content is distributed under the terms of the Creative Commons Attribution 4.0 International license.

### Prediction of viral hosts.

**FIG 7:**
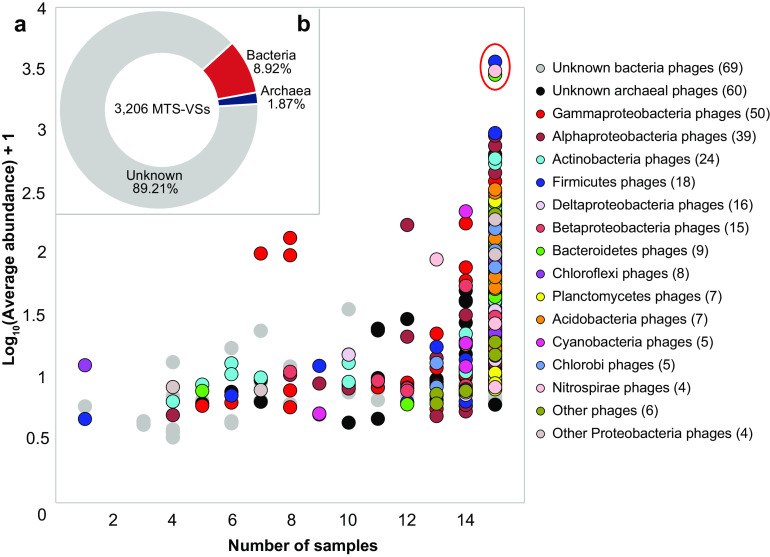
Host prediction of the MTS viruses. (a) Taxonomic affiliation of MTS-VSs sorted by distribution (*x* axis) and average abundance (*y* axis), according to host prediction. Here, the average relative abundance (*y* axis) of an MTS-VS is defined as the mean relative abundance of this viral scaffold across all samples. The numbers of viral scaffolds with predicted hosts of the same phylum are shown in parentheses. The three circles within the red oval represent the most abundant viral scaffolds with predicted hosts, and these exist in all the samples. (b) Proportion of MTS-VSs with predicted hosts.

Only 6.11% of the viral scaffolds (196 out of 3,206) could be assigned to their putative hosts at the phylum level (14 phyla) and the genus level (134 genera). The former were dominated by *Proteobacteria* (mainly *Gammaproteobacteria*), *Actinobacteria*, *Firmicutes*, and *Bacteroidetes*, whereas the latter were dominated by Pseudomonas, *Salinispora*, *Geobacter*, *Bacillus*, *Bradyrhizobium*, *Magnetospirillum*, and *Vibrio* (Fig. 7a; see also Table S3 at https://doi.org/10.6084/m9.figshare.c.5703367.v6). Notably, three viral scaffolds potentially infecting *Firmicutes* (*Clostridium*) species, *Nitrospirae* (*Thermodesulfovibrio*) species, and *Bacteroidetes* (*Aquimarina*) species were present in all the samples with the highest abundances (Fig. 7a). In addition, six viral scaffolds were predicted to be *Salinispora* (*Actinobacteria*) phages, which were rarely reported in the past (38) (see Table S3 at https://doi.org/10.6084/m9.figshare.c.5703367.v6).

## DISCUSSION

At present (i.e., as of November 2021), more than 3.11 million viral sequences have been stored in GenBank. However, despite this, the vast majority of the benthic virus communities from the Mariana Trench sediments in this study lack any homologs in the current databases and are considered unknown novel viruses. Only 6.92% of the MTS viral scaffolds can be annotated with putative taxonomic identifications (see Table S3 at https://doi.org/10.6084/m9.figshare.c.5703367.v6), and more than half of the MTS viral proteins do not have any homologs among all previously identified viral proteins. In addition, these novel viruses have very high diversity, as reflected by the numerous independent branches composed of only the MTS viruses throughout the phylogenetic trees (Fig. 3; see also Fig. S4 in the supplemental material).

We obtained a total of 111 high-quality metagenome-assembled viral genomes. After removing the potential eukaryotic virus genomes, more than 63% of the viral genera represented by these high-quality viral genomes were novel (Fig. 3). Among these novel viral genera, there was a jumbo phage whose genome was ∼217 kb in length (39) (gOTU13 in Fig. 3). A total of 79% of the ORFs in the genome of this jumbo phage were functionally unknown. This virus contains many more genes encoding DNA/RNA polymerase, tRNA, and structural proteins than other viruses (Fig. S9), which is a common feature of jumbo phages (40).

The order *Caudovirales* is the most populous virus order and accounts for approximately 30% of all recognized viral species (International Committee on Taxonomy of Viruses [ICTV], March 2021, https://talk.ictvonline.org/). A similar phenomenon was also observed in this study (Fig. 2d and e) and many other deep-sea sediments (41–44). Besides *Caudovirales*, some MTS viral scaffolds and genes were taxonomically assigned to NCLDVs, which include the families *Phycodnaviridae*, *Marseilleviridae*, *Ascoviridae*, and *Mimiviridae* (Fig. 2d and e). In the upper slope sediments of the Mariana Trench, the most abundant NCLDV scaffolds and genes were from *Phycodnaviridae* viruses affecting eukaryotic phytoplankton (45) (Fig. 2d and e). We also assembled two complete viral genomes of *Phycodnaviridae* affiliated with the same genus (see Fig. S11 at https://doi.org/10.6084/m9.figshare.c.5703367.v6), which contains a pelagophyte virus affecting Aureococcus anophagefferens (46). In fact, the discovery of *Phycodnaviridae* viruses in the sediments of the Mariana Trench is not unusual. The sequences of eukaryotic algal viruses have also been detected in the Baltic Sea subseafloor sediments (47), the sediments of Loki’s Castle hydrothermal vent (∼3,200 m below sea level) in the mid-Atlantic Ocean (29), and the abyssopelagic Yap Trench sediments (48). The presence of phytoplankton viruses might be explained if viruses cosink with eukaryotic phytoplankton from the upper water column via the sinking mechanism of the biological pump (47, 49). This speculation can be further verified by the dominance of phytoplankton-derived organic carbon (4) and the presence of diverse phytoplankton genes (50) in the Mariana Trench sediments and hadal seawater, respectively. We must acknowledge that it was difficult to accurately quantify how many of the identified viral genomes were affiliated with allochthonous or autochthonous viruses. However, we cannot rule out the possibility that even as allochthonous viruses in deep-sea sediments, some members may have the potential to infect their susceptible hosts, as they might maintain their activities in the deep ocean (51).

Based on the results of viral host prediction, we speculate that many benthic viruses in the Mariana Trench are indigenous predators of the prokaryotic groups dwelling in the deep sediments, such as *Salinispora* (52), *Bradyrhizobium* (53), *Nitrospira* (53), and *Woeseia* (54) (see Table S3 at https://doi.org/10.6084/m9.figshare.c.5703367.v6). Typically, six MTS phages were predicted to infect *Salinispora*. *Salinispora* species are globally distributed in marine sediments (52), whereas there has never been any specific information about their phages. Here, we also obtained two *Salinispora* phage genomes with a completeness of >50%; however, since 80% of their ORFs are functionally unknown (see Fig. S10 and Table S7 at https://doi.org/10.6084/m9.figshare.c.5703367.v6), their potential life characteristics are difficult to predict. This highlights the absolute importance of virus isolation and cultivation from the sediments of the Mariana Trench in the future.

Lysogeny is prevalent in approximately half of bacteria in nature (55), and 374 viral scaffolds were predicted as proviruses to infect the dominant bacteria in this study (see Table S3 at https://doi.org/10.6084/m9.figshare.c.5703367.v6). As reported previously, prophages can be protected from decay by lysogeny (55) and likely enhance the survival and competitiveness of bacterial hosts by assisting host metabolism based on the expression of some auxiliary metabolic genes carried by prophages (56) and the prevention of lysogens from subsequent phage infection (57). Thus, in harsh environments, lysogeny might be an effective environmental adaptive mechanism of viruses, contributing to the long-term coexistence of viruses and their hosts in the Mariana Trench sediments.

It has been reported that some of the viral AMGs are expressed during infection, thereby participating in host metabolism and driving biogeochemical cycling in different environments (58). For example, the *psbA* and *psbD* genes in cyanophage genomes can assist with the photosynthesis of their hosts (34). Various auxiliary genes encoding carbohydrate-active enzymes existing in viruses might assist their host in polysaccharide degradation in mangrove sediments (37). Moreover, six AMGs associated with nitrification, nitrate reduction, denitrification, and nitrite transport have been identified in virioplankton metagenomes across the minimum-oxygen zones of the Eastern Tropical South Pacific, and they potentially modulate nitrogen metabolism processes (59). In addition, AMGs involved in sulfur metabolisms, such as sulfur oxidation (60, 61), sulfate reduction (44), and sulfite reduction (62), were also identified in widespread marine viruses. Here, we also observed various viral AMGs that might participate in carbon, nitrogen, and sulfur metabolism in the upper slope sediments of the Mariana Trench. Most of these viral AMGs were speculated to encode functionally active enzymes, as their protein products contained high-confidence structure models and the key functional domains.

The Mariana Trench is like a trap for capturing organic matter (4). Similar to a previous report (4), in this study, we also revealed that the organic matter in the upper slope sediments of the Mariana Trench might be primarily sourced from phytoplankton (22) (see Table S1 at https://doi.org/10.6084/m9.figshare.c.5703367.v6). Glycosides and polysaccharides are among the main components of phytoplankton-derived carbohydrates (4, 63). Interestingly, viral AMGs encoding glycoside hydrolases (GH6, GH9, GH39, and GH87) and a polysaccharide lyase (PL1) that may assist in the degradation of algal cellulose and pectin were observed in the sediments of the Mariana Trench. Phylogenetic analysis indicates that these viral AMGs are likely transferred horizontally from the host bacteria of *Proteobacteria* (*Beta*- and *Gammaproteobacteria*) and *Gemmatimonadetes* (Fig. S6), and some strains of these bacterial groups found in hadal trenches had the potential to degrade cellulose (64, 65). We speculate that in order to better adapt to the trench sediment environment, the viruses may assist their hosts with the degradation of phytoplankton-derived organic matter during the infection process in the extreme benthic environment and thus provide energy for their own reproduction. Meanwhile, the increased prevalence of carbohydrate metabolic genes among viruses is likely due to the increased prevalence of these genes in host genomes in the Mariana Trench sediments.

Except for the viral AMGs involved in organic matter degradation, some viral AMGs potentially participating in dark carbon fixation were also discovered. In fact, autotrophic carbon fixation is an important way for microorganisms to obtain energy in the deep-sea environment (31, 66). As reported previously, dark chemoautotrophy rates range from 0.03 to 10.37 μmol C m^−3^ day^−1^ in the North Atlantic (67), and the chemoautotrophy in the dark ocean amounts to 15 to 53% of the export-production of phytoplankton (68). Here, the functional genes (e.g., PPDK and ACC) in viral genomes potentially participating in the main pathways (rTCA, DC/4-HB, 3-HP, and 3-HP/4-HB cycles) (31) of inorganic carbon fixation were discovered, and structural prediction suggests that the encoded products PPDK and ACC are functionally active (Fig. 4b). To the best of our knowledge, a viral AMG encoding ACC has never been reported in marine viruses. It is worth mentioning that although the PPDK and ACC proteins are not the key enzymes in carbon fixation pathways, we cannot rule out that they may participate in the dark carbon fixation process to improve the energy metabolic efficiency of their hosts. This hypothesis needs to be further verified in the future and may represent a previously unknown survival strategy for viriobenthos in deep sediments.

The MTS viruses also contained an AMG involved in denitrification (see Table S9 at https://doi.org/10.6084/m9.figshare.c.5703367.v6). In detail, the circular viral genome of “VIRSorter_k141_16118572_length_105477_cov_18_9918-circular-cat_2” contains a *nosZ* gene potentially participating in the reduction of N_2_O to N_2_. An ammonium transporter gene was also present in this viral genome (Fig. 5d). Notably, 68% of the annotated proteins of this viral scaffold were homologous to their counterparts in the archaeal phylum *Thaumarchaeota*, including these two nitrogen metabolic genes (Fig. 5a and b; see also Table S5 at https://doi.org/10.6084/m9.figshare.c.5703367.v6), which indicates that they were likely transferred from *Thaumarchaeota*. Indeed, the phylum *Thaumarchaeota* is the most dominant archaeal group in the sediments of the Mariana Trench (Fig. S2a). The distinctive feature of *Thaumarchaeota* regarding function is their capability for ammonium oxidation (69) and denitrification (i.e., converting NO to N_2_O) (70). As mentioned above, the *nosZ* gene can participate in the reduction of N_2_O to N_2_. Viruses containing the *nosZ* gene have the potential to participate in the denitrification process in the sediments of the Mariana Trench.

In addition, various auxiliary sulfur metabolic genes have recently been discovered in viral genomes (61). Here, viral *cysH* and *cysK* genes were discovered in the sediments of the Mariana Trench (Fig. 6 and Fig. S8). Among them, the CysH enzyme can participate in assimilatory sulfate reduction, which is necessary for the synthesis of cysteine and methionine (33, 71). Methionine is the substrate for the bacterial biosynthesis of dimethylsulfoniopropionate (DMSP) (72), which has the highest concentration in the Mariana Trench sediments at a bathymetric depth of ∼6,000 m to protect bacteria against the extremely high hydrostatic pressure (6). Whether the viruses will assist the host in adapting to the high-hydrostatic-pressure environment in the Mariana Trench requires more in-depth studies in this area in the future.

Finally, it is worth mentioning that we did not perform prior virion separation from the Mariana Trench sediments for viromic analysis in this study. Considering that the recovery rate of virus particles separated from sediments is usually low and virion separation will usually neglect those viruses that are in the lysogenic cycle (either integrated into the host genome or as a plasmid), tightly attached to large particles, and those with a diameter of >0.22 μm (43, 73), despite that the accuracy of virus identification by using direct metagenomic analysis of sediments is not as high as the method of preseparation of virus particles from sediments, direct metagenomic analysis of sediment might have the advantage of showing a more complete picture of virus diversity in sediments (43). In addition, with the rapid development of bioinformatics, more and more advanced tools have emerged, such as VirSorter2 (74) and DeepVirFinder (75) for viral identification, VirHostMatcher-Net (76) for viral host prediction, and VPF-Class (77) for viral taxonomic assignment. Future research using these newly developed tools may provide a fuller picture of the viral communities.

In conclusion, the diverse novel viruses and the various viral AMGs that potentially contribute to organic carbon degradation, inorganic carbon fixation, denitrification, and assimilatory sulfate reduction provide novel insight into the composition of benthic viral communities in the upper slope sediments of the Mariana Trench.

## MATERIALS AND METHODS

### Sample collection.

Eight push-core sediment samples were collected using the deep-sea human-occupied vehicle (HOV) *Jiao Long Hao* from eight locations (A, B, D114, D119, D120, D144, D146, and D147) along the northern and southern slopes of the Challenger Deep in the Mariana Trench during cruises DY37II and DY38III in 2016 and 2017, respectively (Fig. 1). Information about longitude/latitude and sampling depth was recorded during field sampling. In addition, onboard, each core was cut into 2-cm-wide sections, and each section was then divided into two fractions. One fraction was immersed in RNAlater (Ambion, Carlsbad, CA) and stored at −80°C until metagenomic analysis. The other fraction was stored at −80°C directly for further physicochemical analyses. Back in the laboratory, the 0- to 2-cm, 2- to 4-cm, and 4- to 6-cm core sections were combined and called 0 to 6 cm (surface part [s]), and those from the 6- to 12-cm (middle part [m]) and 12- to 18-cm (bottom part [b]) sections were generated similarly, for subsequent analysis of their metagenomes and environmental parameters. The physicochemical parameters were also measured in the laboratory. For more information about sediment property analysis and the enumeration of viruses and prokaryotes, see Text S1 in the supplemental material.

10.1128/msystems.01358-21.1 TEXT S1 Detailed methods for sediment property analysis, enumeration of viruses and prokaryotes, microbial community analysis, and phylogenetic analysis of the MTS-VSs. Download Text S1, DOCX file, 0.03 MB. Copyright © 2022 Zhao et al. 2022 Zhao et al. https://creativecommons.org/licenses/by/4.0/ This content is distributed under the terms of the Creative Commons Attribution 4.0 International license.

### DNA extraction, sequencing, and metagenomic analysis.

For DNA extraction, total nucleic acids were extracted from each sediment sample (5 to 15 g) using a FastDNA spin kit for soil (MP Biomedicals, Irvine, CA) according to the manufacturer’s instructions. The final DNA concentrations were measured using a Nanodrop spectrophotometer. Finally, the total nucleic acid yield of 15 samples was extracted successfully and used for subsequent library construction and sequencing (see Table S2 at https://doi.org/10.6084/m9.figshare.c.5703367.v6).

The construction of the DNA library and high-throughput sequencing of the extracted DNA were carried out with an Illumina HiSeq2500 PE150bp platform and the HiSeq cluster kit v4 (Illumina, San Diego, CA, USA) to generate 150-bp paired-end reads. Paired-end reads were trimmed by Trim-galore v0.5.0 (https://github.com/FelixKrueger/TrimGalore) with default settings to remove adapter sequences and low-quality sequences (Phred score of <20). The resulting high-quality reads from each MTS sample were assembled with metaSPAdes v3.13.0 (78) using default parameters. Coassemblies of pooled reads from the 15 samples were also performed to improve the genomic representation of viruses using metaSPAdes. Following assembly, all assembled scaffolds (consisting of contigs separated by gaps) generated by metaSPAdes were used for further virus identification and clustering.

See Text S1 for information about the microbial metagenome (especially the prokaryotic community) analysis.

### Identification and clustering of viral scaffolds.

In our metagenomic data set, the viral sequences probably originate from intracellular and extracellular lytic phages and temperate phages that integrate into the microbial genomes or exist as extrachromosomal elements. All scaffolds affiliated with viruses were identified and clustered according to a method reported previously (79) but with minor modifications. In brief, each of the 16 (co)assemblies (15 individual assemblies and 1 coassembly) was individually searched for viral scaffolds according to the following criteria. Scaffolds of ≥1.5 kb were piped through VirSorter v1.0.5 (80) and VirFinder v.1.0.0 (81) to predict the viral sequences. VirSorter is reliable software to determine viral sequences, especially for longer sequences with the hallmark viral genes (80). VirFinder is software that relies on *k-*mer signatures to predict viral sequences (81). Scaffolds (≥5 kb or ≥1.5 kb and circular), which were sorted with a VirFinder score of ≥0.7, a *P* value of <0.05, and/or VirSorter categories 1 to 6, were pooled for further analysis. Those scaffolds satisfying any one of the following conditions were then classified as being viral scaffolds: (i) VirSorter categories 1 and 2; (ii) a VirFinder score of ≥0.9 and a *P* value of <0.05; and (iii) VirSorter categories 1 to 6, a VirFinder score of ≥0.7, and a *P* value of <0.05. Any remaining scaffolds with <40% (based on an average gene size of 1,000) of the scaffold classified as bacterial, archaeal, or eukaryotic with the sequence annotation tool CAT v4.6 (82) were also considered viral scaffolds. The viral scaffolds obtained from 16 (co)assemblies were then merged and grouped into populations if they shared ≥95% nucleotide identity across ≥80% of the scaffold length using the BLASTn program in BLAST+ v2.10.1 (83, 84).

For each viral population, the open reading frames (ORFs) were predicted using Prodigal (85), and the resulting protein sequences were loaded into vConTACT2 v0.9.10 (86) to cluster the MTS viruses and known viruses from the viral RefSeq database (release 96) with parameters -rel-mode Diamond, -pcs-mode MCL, and -vcs-mode ClusterONE. Based on the protein-sharing network constructed by vConTACT2, MTS viral populations and known viruses were grouped into viral clusters. For those viral clusters containing MTS viral scaffolds, the longest MTS viral scaffold of each viral cluster was selected and merged to assemble a Mariana Trench sediment viral scaffolds (MTS-VSs) data set consisting of 3,206 unique viral scaffolds (all ≥5 kb, at the subfamily/genus level). The completeness of viral genomes represented by viral scaffolds was estimated using CheckV v0.7.0 (87).

### Taxonomic assignments and abundance profiles of viral scaffolds.

Those viral scaffolds clustered with a virus from RefSeq by vConTACT2 were able to be assigned to a known viral taxonomic genus and family (79). Besides, BLAST analysis of viral scaffolds was also performed against the Integrated Microbial Genome/Virus (IMG/VR) system v.3.0 database (88) (BLASTn E value of ≤10^−5^, similarity of ≥90%, and covered length of ≥75%) to assign the taxonomies for viral scaffolds based on sequence similarity (49, 83). Additionally, a majority-rules approach was performed for the taxonomic assignments as previously described (48, 88). Briefly, predicted proteins from viral scaffolds were compared to NCBI viral RefSeq proteins (release 203) using DIAMOND v2.0.4 (89) (BLASTp; -subject-cover 50; –more-sensitive; -query-cover 50; -evalue 1e-5), and a viral scaffold was assigned to a taxonomy if ≥50% of the proteins were assigned to that taxonomy. Besides, CAT v5.0 (82) and ViralRecall v2.0 (-s 2) (28) were used to taxonomically classify viral scaffolds and detect nucleocytoplasmic large DNA viruses (NCLDVs), respectively. To verify the novelty of MTS viruses, predicted proteins from MTS-VSs were compared to the IMG/VR protein database v3.0 using DIAMOND v2.0.4 (BLASTp; E value of <1e−5, identity of >30%, and coverage of >50%).

Metagenomic reads of all samples were mapped to all MTS-VSs and all predicted viral genes to calculate the relative abundances of viral scaffolds and genes, respectively, using the BBMap package v38.86 (https://sourceforge.net/projects/bbmap/) (49). The read counts were normalized based on the transcripts per million (TPM) calculation (90) by BBMap, and the generated abundance matrix was thereafter used as an input for diversity analyses.

### Phylogenetic analysis of viral scaffolds.

Phylogenetic trees of MTS viruses were generated based on the high-quality viral genomes (completeness of >90%) and a viral group-specific marker, the *terL* gene for *Caudovirales* (91). The proteomic trees of all the high-quality viral genomes were constructed as previously described but with minor modifications (30). The reference viral genomes homologous (all-against-all genomic similarity score [*S_G_*] of >0.15) to at least one of the MTS high-quality viral genomes were obtained from the Virus-Host DB (92) and IMG/VR 3.0 (88) databases, and the all-against-all distance matrix (calculated based on *S_G_* values, 0 ≤ *S_G_* ≤ 1) of the MTS viral genomes and selected reference viral genomes was used to build proteomic trees with BIONJ using ViPTreeGen v1.1.2 and the ViPTree online server (93). We used a threshold of an *S_G_* of >0.15 to cluster the branches into viral gOTUs, as proposed previously (30). For the *terL* gene phylogenetic analysis, all proteins predicted as the phage terminase large-subunit domain (TerL; Terminase_6; PF03237) were retrieved from MTS viral proteins and IMG/VR proteins (v3.0) (88). MTS viral TerL amino acid sequences were compared to those from the IMG/VR database using BLASTp (E value of <10^−5^) to recruit relevant reference sequences. All sequences were aligned and trimmed, and the maximum likelihood tree was constructed using IQ-tree v1.6.12 (94). The trees were then visualized with iTOL v6 (95). A more detailed description of this protocol is provided in Text S1.

### Host predictions and provirus identification.

The potential microbial hosts of the benthic viruses found in the sediments of the Mariana Trench were predicted by PHISDector (released 14 July 2020) (96) using its default parameters. This software was used *in silico* based on the multiple phage-host interaction signals (PHISs), including prophages, oligonucleotide profile/sequence composition, alignment-based similarity, CRISPR targeting, protein-protein interactions, cooccurrence/coabundance patterns, and special host-related gene (i.e., virulence factor gene and antibiotic resistance gene) checks (96). Only one best microbial species was selected as the putative host of each viral scaffold based on the overall score or the most matches in the result file. In addition, tRNA sequences were retrieved and queried against the NCBI nr database of bacterial genomes using BLASTn, and only the best hits (i.e., with a minimum of 90% coverage and 90% identity) were considered putative hosts (19). Finally, these results of host prediction were combined and curated manually as previously reported (44, 97).

Provirus-like viral scaffolds were predicted by BLAST analysis against the microbial bins (BLASTn E value of 10^−3^, 70% similarity, and 75% of the viral scaffold length) (98) and using PHISDector (96), CheckV v0.7.0 (87), and VIBRANT v1.2.1 (99). In addition, viral scaffolds harboring lysogenic marker proteins, such as integrase, excisionase, invertase, serine recombinase, and CI/Cro repressor (48, 100, 101), are considered potential proviruses.

### Functional profiles and identification of AMGs.

All virus-encoded proteins were queried against the eggNOG database (102) using a python script, emapper.py v1.0.3, with the parameters -m diamond and –seed_orthology_evalue 0.00001 (103), and the COG information for each protein was assigned. The functional profiles were determined for each sample by summing up the genetic abundances of the proteins belonging to the same COG.

The AMGs of MTS-VSs were identified as previously reported but with minor modifications (44). In detail, AMG annotations were performed using VIBRANT v1.2.1 (99) based on the KEGG, Pfam, and VOG annotations of each viral protein using default parameters. Specifically, the genes with the KEGG annotation “energy metabolism” were selected as the putative AMGs. In addition, viral scaffold annotations were also performed using DRAM-v, the viral model of DRAM v.1.2.0 (104). First, based on VirSorter v2.0 (74), 2,449 viral scaffolds were selected for further DRAM-v annotation due to their high viral scores (>0.5) (74), and the needed input files of DRAM-v were produced. Next, the selected viral scaffolds were run through DRAM-v using default parameters, and those genes with gene descriptions and auxiliary scores of <4 were considered putative AMGs. As suggested previously (44, 105), to be conservative, we did not consider the genes related to glycosyltransferases, ribosomal proteins, organic nitrogen, and nucleotide metabolism as viral AMGs. For all putative AMGs annotated by VIBRANT and DRAM-v and to avoid false-positive results for selected AMGs caused by possible pollution of host sequences, only the putative AMGs located between two viral hallmark genes or virus-like genes and those located alongside the viral hallmark genes or virus-like genes were considered high-confidence viral AMGs for further analysis. For the viral scaffolds containing AMGs, genes without annotations by VIBRANT and DRAM-v were searched against the NCBI nr and UniProt databases using BLASTp with an E value threshold of 0.001, an identity of 30%, and coverage of 50%. Amino acid sequences of AMGs were inputted into the Phyre2 (v2.0) Web portal to search the protein structure homology, and the predicted three-dimensional structures of viral proteins were obtained online (106). Conserved domains of viral AMGs were identified using the NCBI CD-search tool (107). Genome maps for AMG-containing viral scaffolds were visualized based on VIBRANT, DRAM-v, and VirSorter2 annotations.

It was previously shown that viral AMGs can be obtained and maintained from their hosts (34), so AMG phylogeny is an alternative method for predicting putative hosts (35–37). The relevant reference sequences of the relevant species were retrieved by comparing the amino acid sequences of AMGs against the nr database (online comparison) (BLASTp E value of ≤10^−5^ and bitscore of >50) (35, 37). The amino acid sequences of the reference sequences and AMG sequences were used to establish an AMG phylogenetic tree via the GGDC Web server (108), which is available at http://ggdc.dsmz.de/, using the DSMZ phylogenomics pipeline (109) adapted to single genes. iTOL v6 was also used to edit the trees manually.

### Statistical analysis.

The R packages vegan (110) and ape (111) were used to estimate the alpha diversity indexes (Chao1 and Shannon) and perform PCoA, ADONIS, NMDS analysis, and ANOSIM of the viral and prokaryotic communities. Regression analyses and their visualization were performed using the R packages lme4 (112) and ggplot2 (113). Correlation analysis was performed using the cor.test function in R. Heat maps, box plots, and scatterplots were drawn using the ImageGP online tools (EHBio). Distance-based redundancy analysis (db-RDA) was performed using the R packages vegan (110) and rdacca.hp (114) to interrogate the significant environmental variables affecting the viral and prokaryotic communities.

### Data availability.

The quality-controlled reads for 15 metagenomes are stored in the NCBI Sequence Read Archive (SRA) with accession numbers SRR11659604 to SRR11659618 under BioProject accession number PRJNA629672 (see Table S2 at https://doi.org/10.6084/m9.figshare.c.5703367.v6) and the National Omics Data Encyclopedia (NODE) (https://www.biosino.org/node/index) with the accession number OEP001681. All the nucleotide sequence data for the viral scaffolds reported here were deposited in the Genome Warehouse in the National Genomics Data Center (115), Beijing Institute of Genomics (China National Center for Bioinformation), Chinese Academy of Sciences, under accession number GWHASIT00000000, and they are publicly accessible at https://bigd.big.ac.cn/gwh.
