# Supplementary figures and images for: Novel Viral Communities Potentially Assisting in Carbon, Nitrogen, and Sulfur Metabolism in the Upper Slope Sediments of Mariana Trench
Source: mSystems. 2022 Jan 4;7(1):e01358-21. doi: 10.1128/msystems.01358-21 (PMC8725595; doi:10.1128/msystems.01358-21)

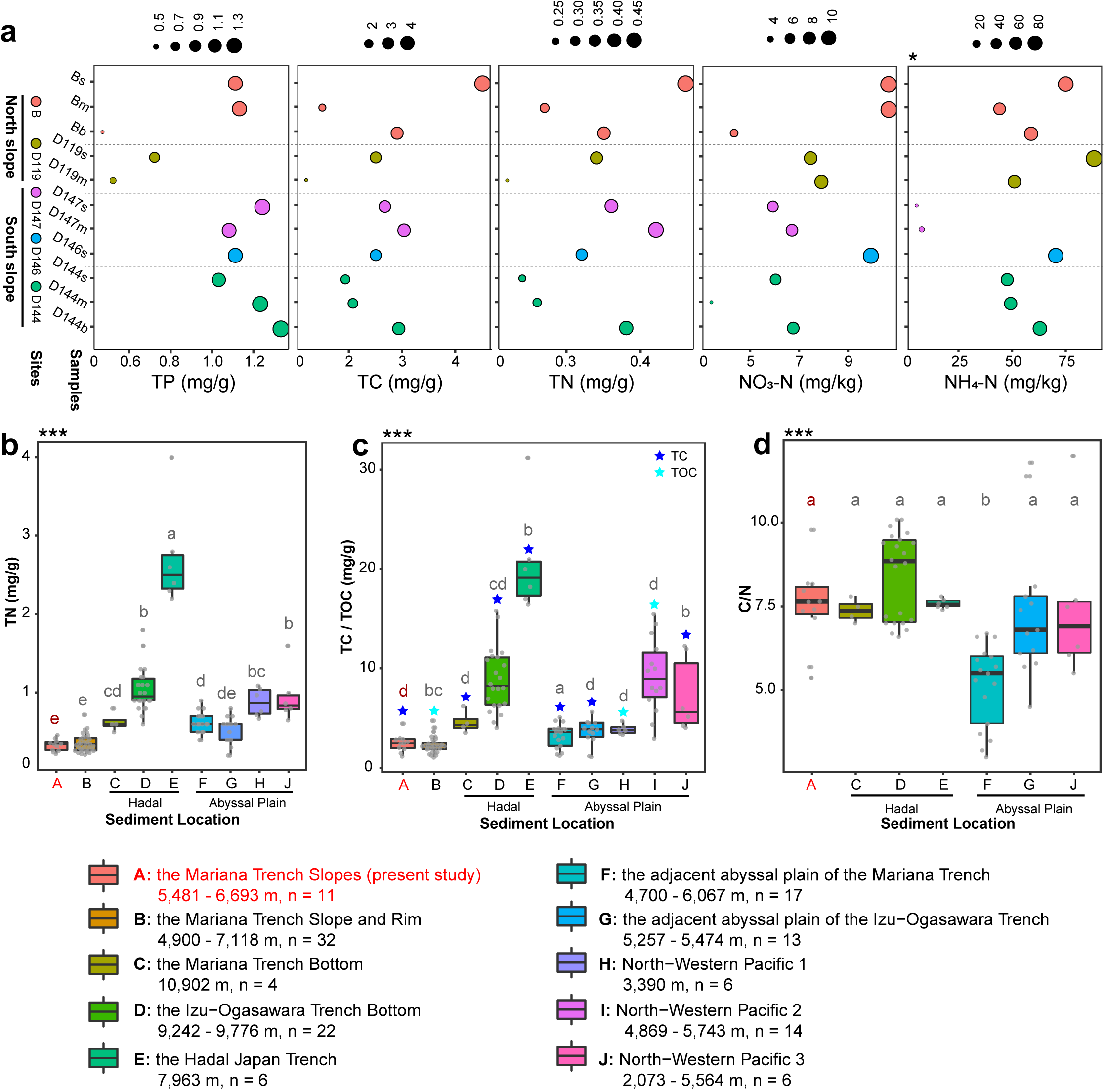

Supplement: FIG S1 [file msystems.01358-21-sf001.tif]

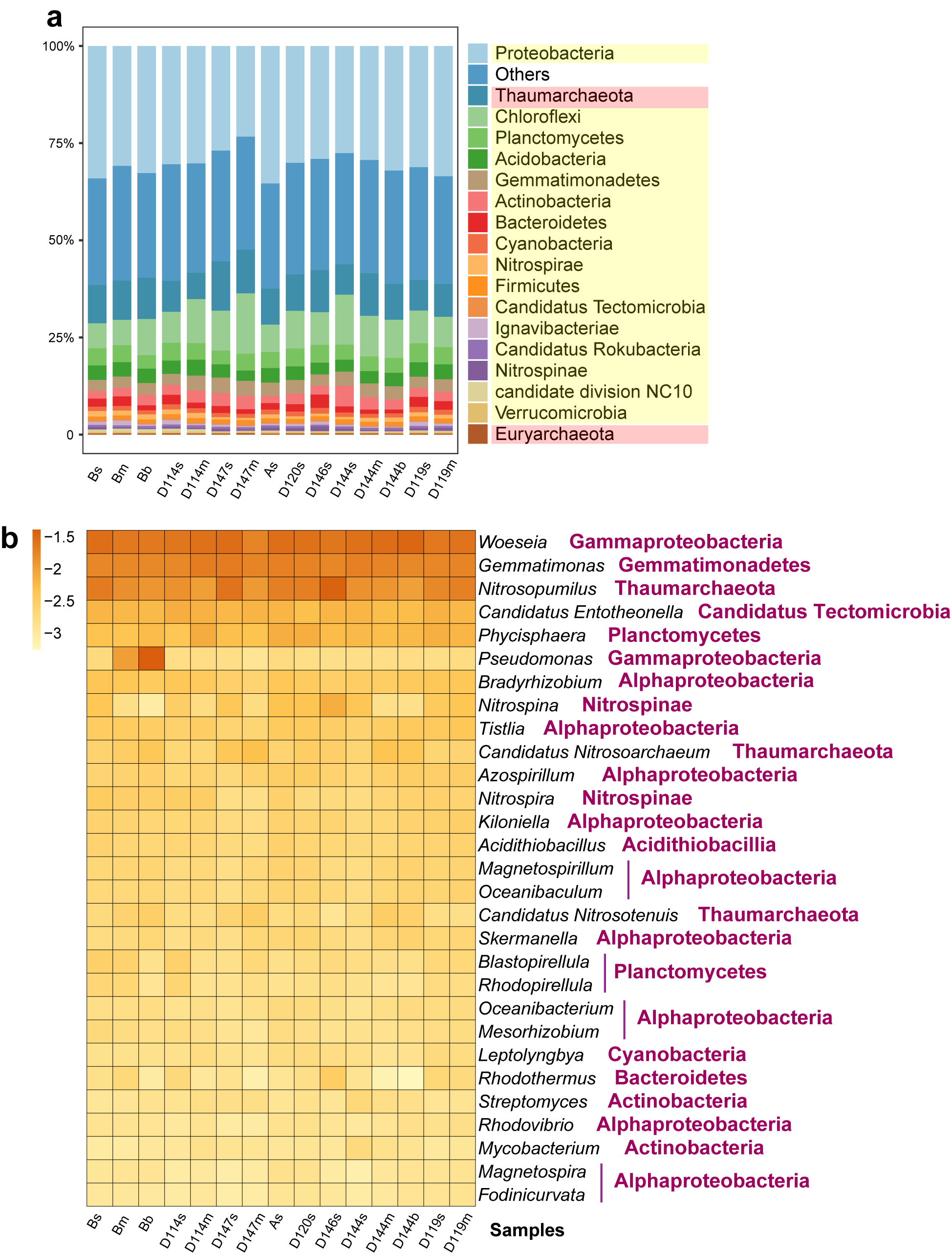

Supplement: FIG S2 [file msystems.01358-21-sf002.tif]

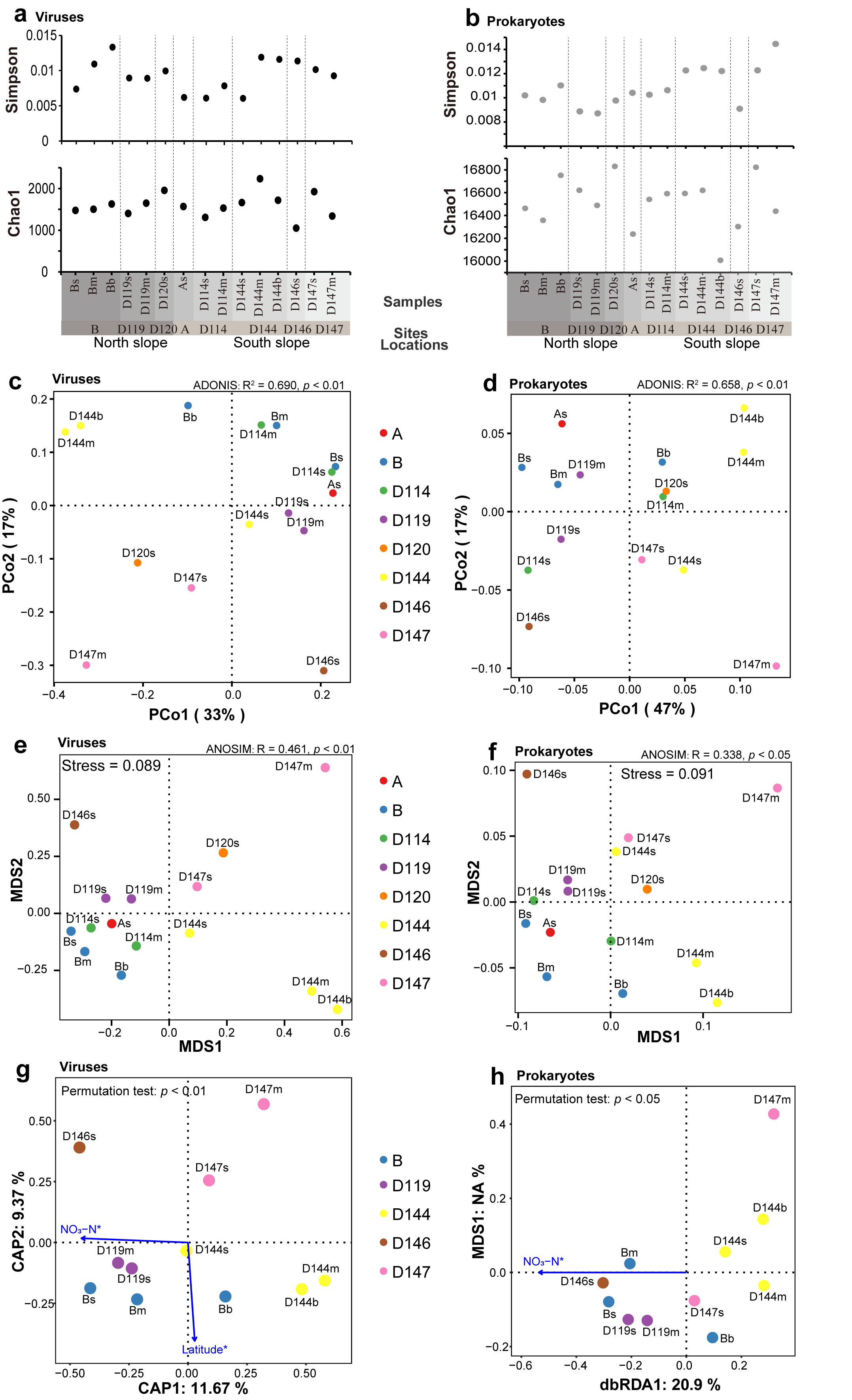

Supplement: FIG S3 [file msystems.01358-21-sf003.tif]

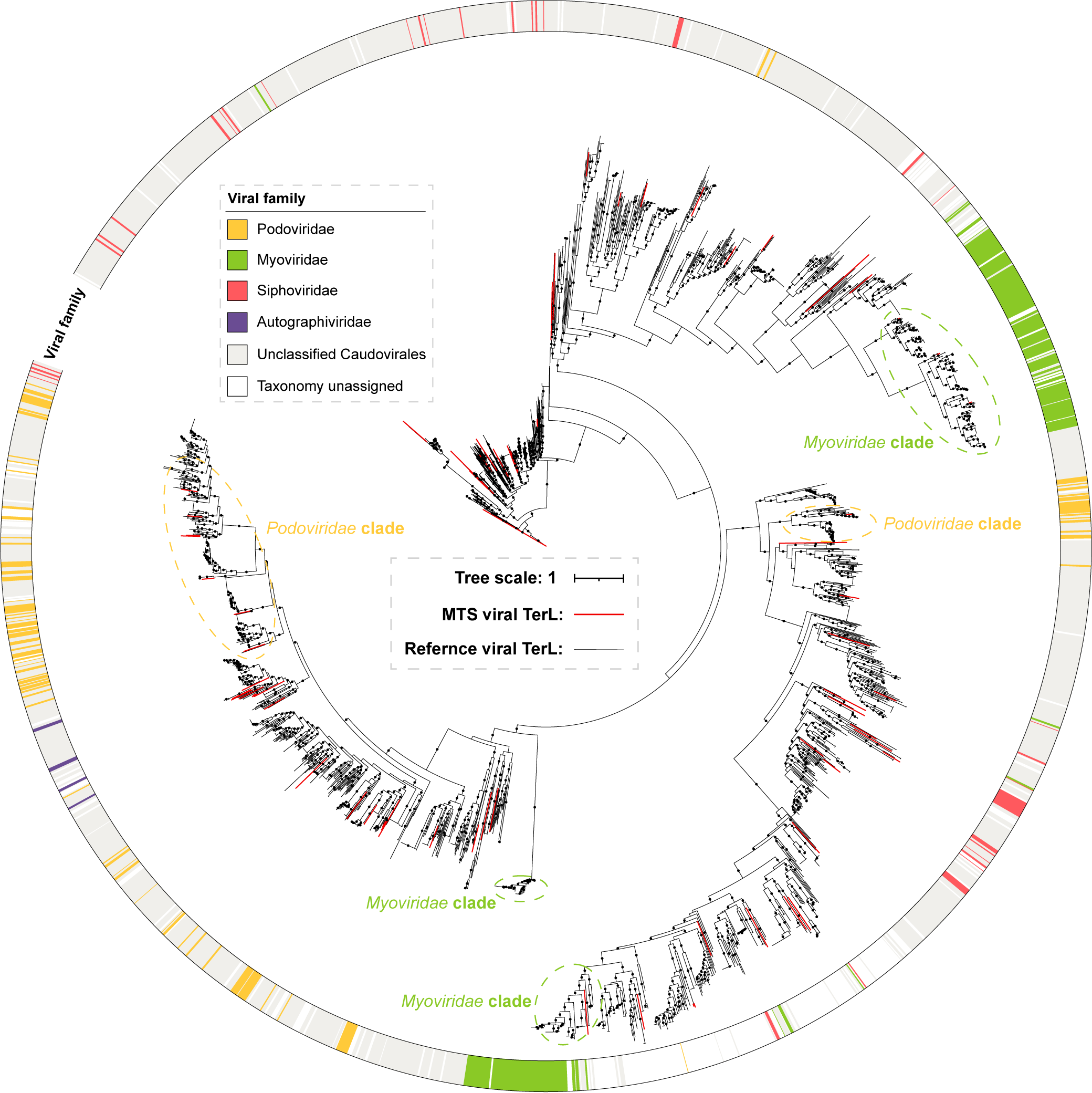

Supplement: FIG S4 [file msystems.01358-21-sf004.tif]

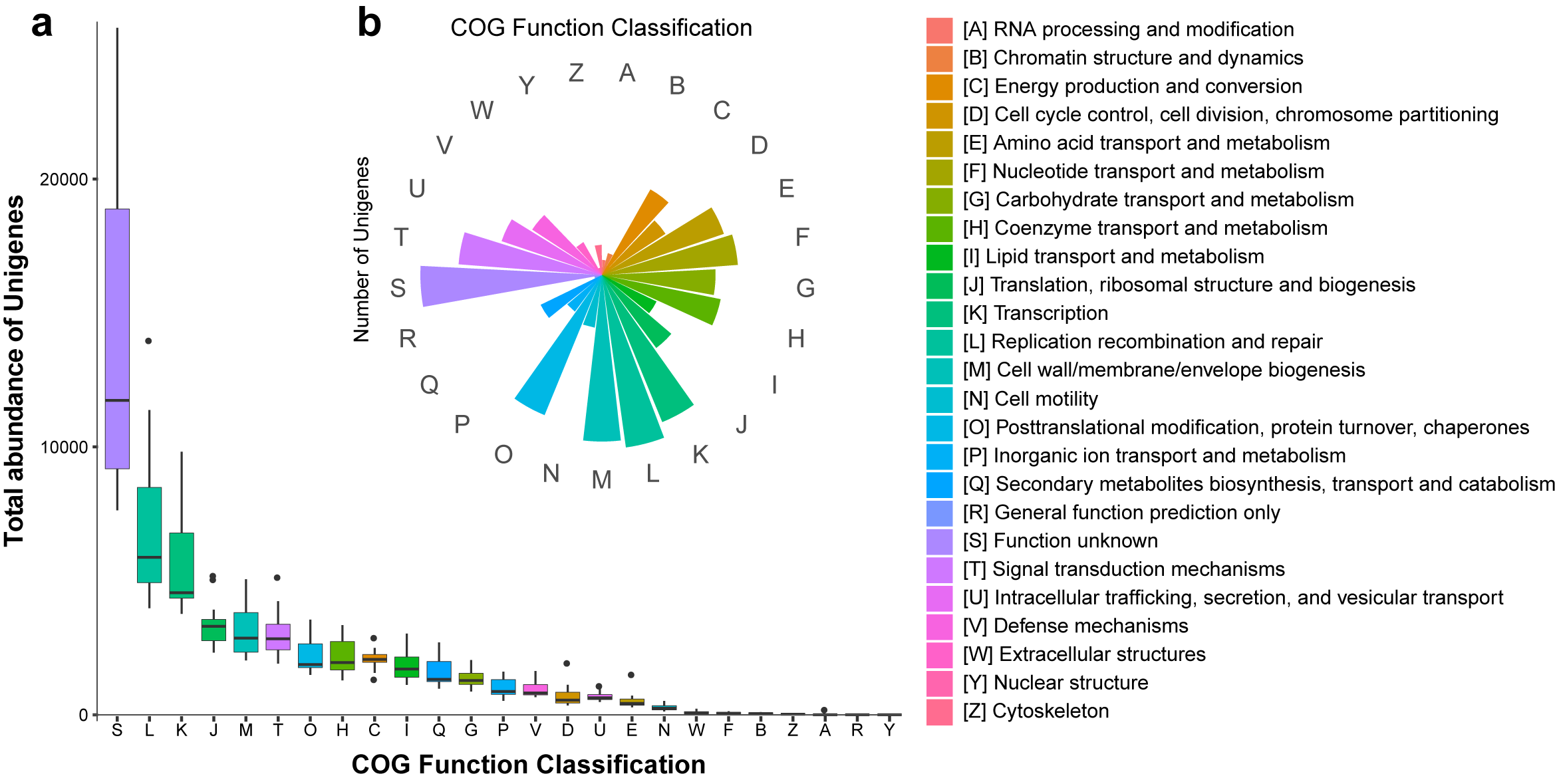

Supplement: FIG S5 [file msystems.01358-21-sf005.tif]

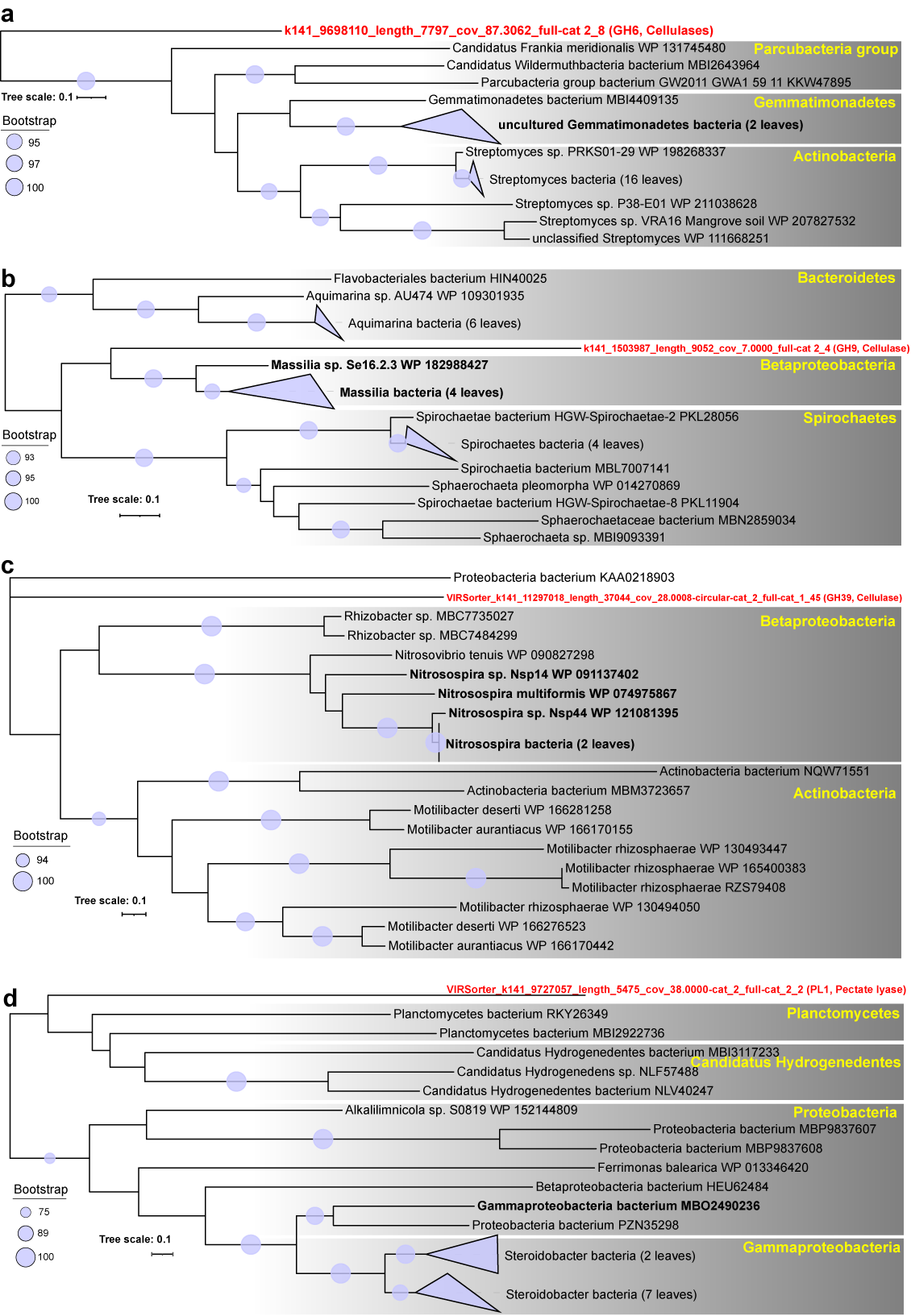

Supplement: FIG S6 [file msystems.01358-21-sf006.tif]

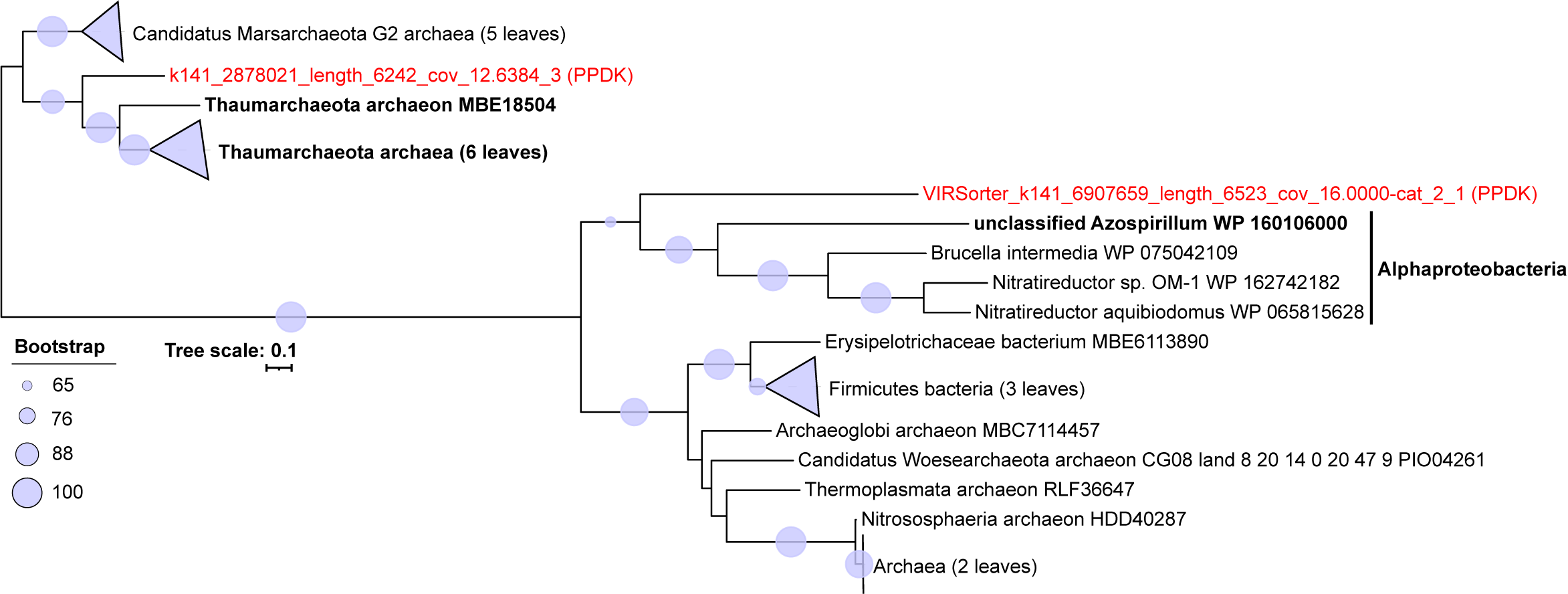

Supplement: FIG S7 [file msystems.01358-21-sf007.tif]

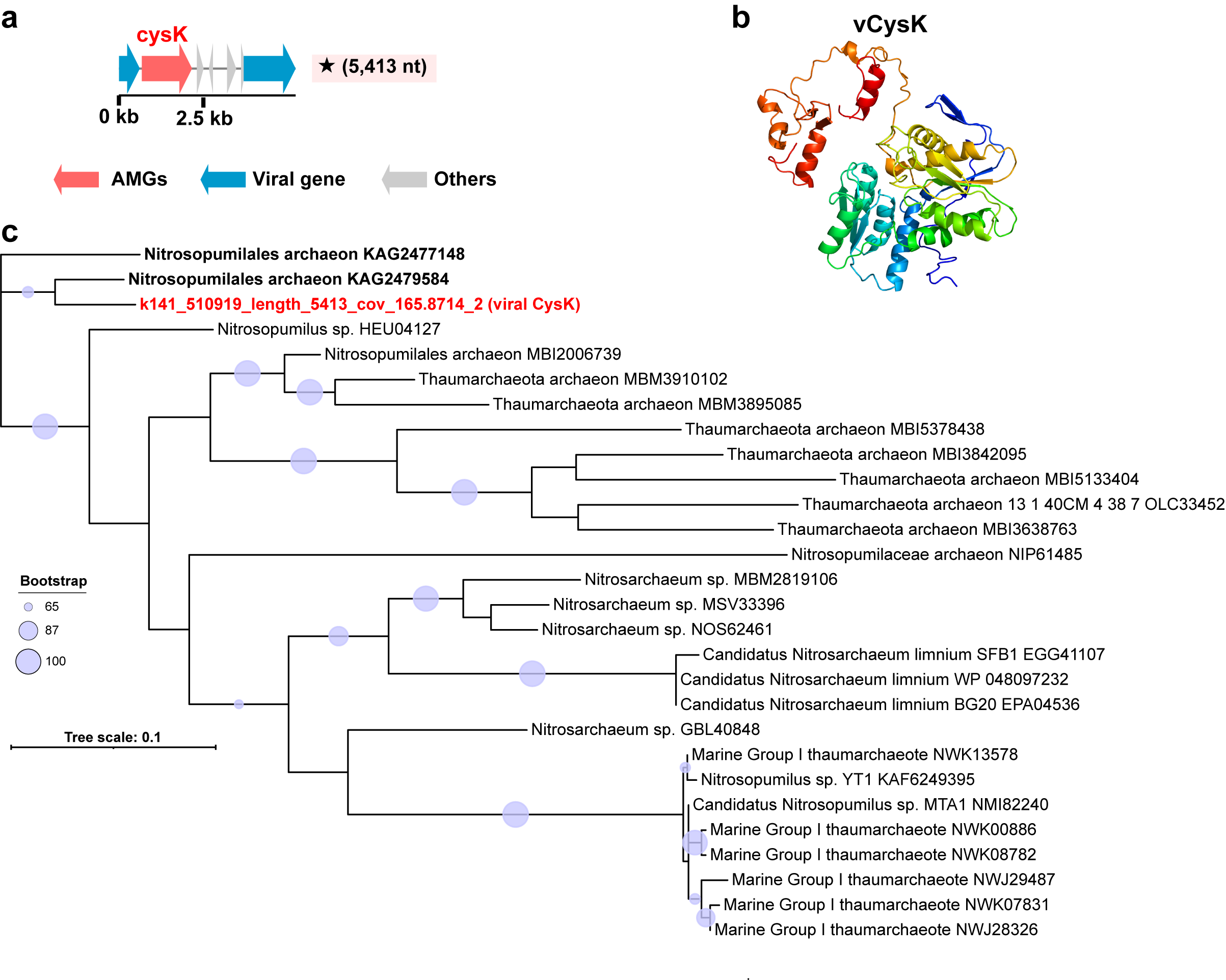

Supplement: FIG S8 [file msystems.01358-21-sf008.tif]

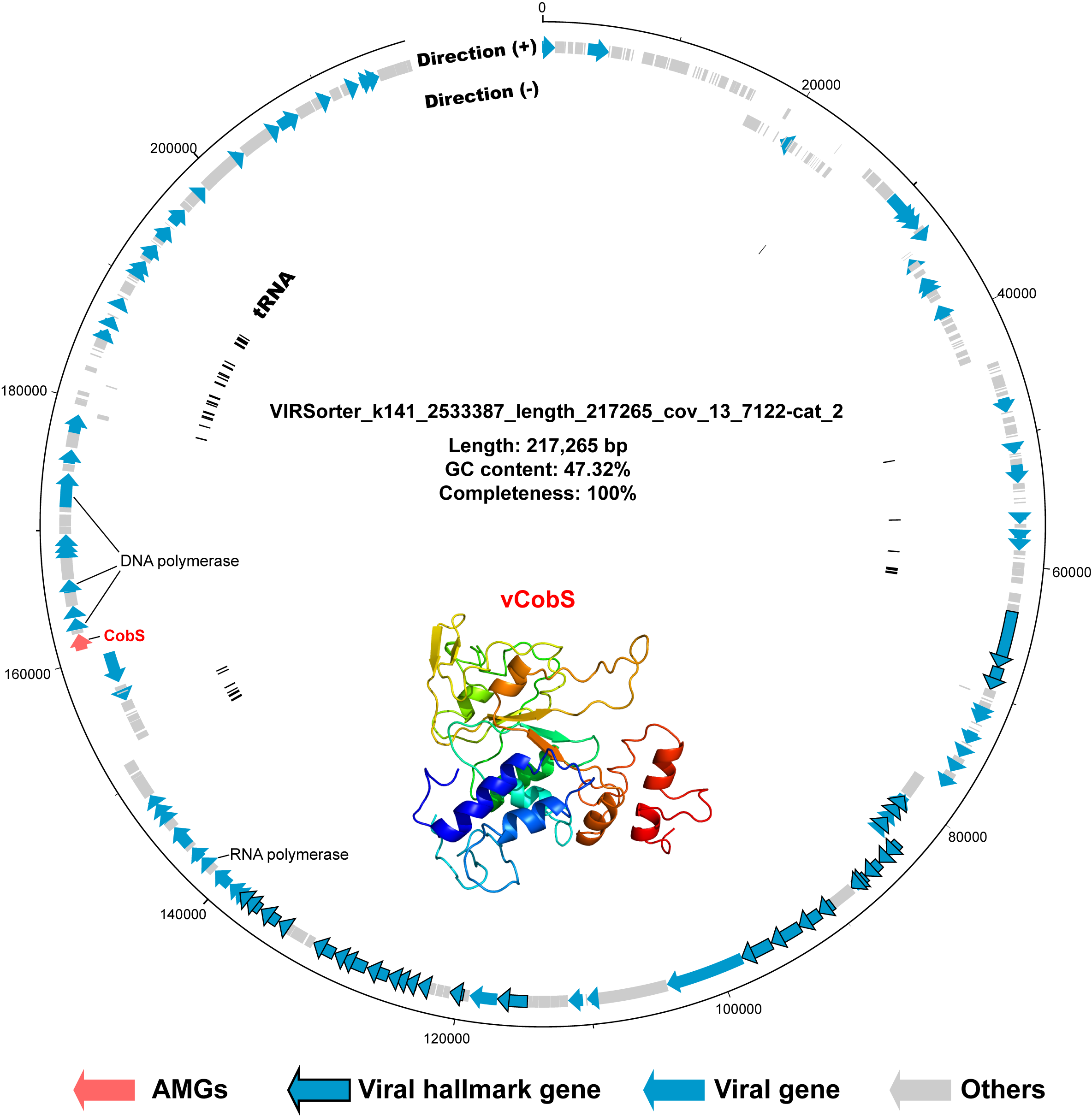

Supplement: FIG S9 [file msystems.01358-21-sf009.tif]
