# Supplementary material for: Novel Viral Communities Potentially Assisting in Carbon, Nitrogen, and Sulfur Metabolism in the Upper Slope Sediments of Mariana Trench
Source: mSystems. 2022 Jan 4;7(1):e01358-21. doi: 10.1128/msystems.01358-21 (PMC8725595; doi:10.1128/msystems.01358-21)
Supplement: TEXT S1 [file msystems.01358-21-s0001.docx]

Supplemental Text for

**Novel viral communities potentially assisting in carbon, nitrogen, and sulfur metabolism in the upper slope sediments of Mariana Trench**

Jiulong Zhao^1,3^, Hongmei Jing^2,3*^, Zengmeng Wang^1,3^, Long Wang^1,4^, Huahua Jian^5^, Rui Zhang^4^, Xiang Xiao^5^, Feng Chen^6^, Nianzhi Jiao^4^, Yongyu Zhang^1,3*^

*Corresponding Authors.

E-mail: Y.Z. zhangyy@qibebt.ac.cn; H.J. hmjing@idsse.ac.cn;

**Supplemental Materials and Methods**

***Sediment properties analysis***

Physicochemical analyses of sediment samples (*ca.* 5 g) were conducted at the Institute of Mountain Hazards and Environment, Chinese Academy of Sciences (Chengdu, Sichuan, China), according to a previous report (1). In brief, nitrate and ammonia were detected after treatment with 1 M HCl, followed by analysis with a colorimetric auto-analyzer (SEAL Analytical AutoAnalyzer 3, Germany). The concentrations of total carbon (TC) and total nitrogen (TN) were determined by over-drying the sediments at 105°C and then using an element analyzer (Elementar vario Macro cube, Germany). Total phosphate (TP) was measured after digestion of the sediment with nitric-perchloric acid (2), using the molybdate colorimetric method with a UV2450 (Shimadzu, Japan). The wet and dry weights of each sample were recorded before and after being heated in a conventional oven, and the moisture content was calculated as a ratio of weight difference over wet weight (3). All these measurements were carried out in triplicates and the standard error was less than 1%.

The image of the sampling location (Fig. 1a) was generated using Ocean Data View (ODV) (4). The topographic map containing all the sampling sites and bathymetric depths (Fig. 1a) was drawn by the Generic Mapping Tools (GMT) v6 (5).

The TN, TC/TOC, and C/N values of the different sediments were retrieved from the present study, previous studies (6-9), and the IODP dataset (see Table S2 at https://doi.org/10.6084/m9.figshare.c.5703367.v6), and they were illustrated using the bubble chart and box and jitter plots shown in Fig. S1 via ImageGP online tools (http://www.ehbio.com/ImageGP/index.php/Home/Index/index.html).

***Enumeration of viruses and prokaryotes***

The viral and prokaryotic abundances were determined by epifluorescence microscopy as previously reported but with minor modifications (10). Duplicate 0.5-1 g (wet weight) samples of frozen sediments were diluted with 4.25 ml virus-free seawater (pre-filtered through 0.02-μm-pore-size filters; Whatman Anodisc) and homogenized by vortexing for 1 min. To separate the viruses and prokaryotes from the sediment particles, the slurries were incubated with 5 mM (final concentration) sodium pyrophosphate solution (0.02 μm filtered) and left to stand for 15 minutes in the dark after mixing thoroughly. Then the ultrasonic treatment was performed at 100W and 47kHz for 3 minutes with 30 seconds manual shaking every minute. The mixtures were serially diluted 500-fold with virus-free seawater and centrifuged at 800 × g for 2 min to reduce the number of suspended particles. The supernatant was removed and incubated with DNase I (2 U/ml) for 15 min in the dark at room temperature to remove extracellular DNA. Five milliliters of the supernatant were then filtered onto 0.02 µm pore size filters (Anodisc Al_2_O_3_, 25 mm diameter). The filters were stained with SYBR Gold and mounted to a microscopic slide using 60 μl of anti-fade solution [PBS (0.05 mol/l Na_2_HPO_4_ and 0.85% (wt/vol) NaCl):glycerol (1:1) and 0.1% p-phenylenediamine] after rinses with 1 ml virus-free MilliQ water on the back of the filters. The slides were viewed with a fluorescence microscope (Leica DM2000 LED) under blue light (wavelength: 365 nm). Virus-like particles (VLPs) and cells were manually counted from more than 30 microscopic fields, ensuring a minimum count of 400 for each sample. Finally, the virus and prokaryote abundances of dry weight sediments were calculated with the water content.

***Microbial community analysis***

The microbial community analysis was carried out by Novogene Company (Beijing, China), and the analysis pipeline is as follows. Clean reads from each MTS sample were assembled with SOAPdenovo2 (K-mer=55) (11) individually. Unmapped reads to the assemblies were collected using Bowtie2 (--end-to-end, --sensitive, -I 200, -X 400) (12) and merged together to implement the cross-sample assemblies with SOAPdenovo2 (K-mer=55) (11). All assembled scaffolds were interrupted from the gap to obtain the scaftigs (i.e., continuous sequences within scaffolds) (13), and those scaftigs < 500 bp were filtered. Among all scaftigs, open reading frame (ORF) prediction was performed by MetaGeneMark (default parameter) (14), and the redundant predicted ORFs were removed by CD-HIT (-c 0.95, -G 0, -aS 0.9, -g 1, -d 0) (15) to obtain the unique gene catalogue. Clean reads of all samples were mapped to the unique gene catalogue using Bowtie2 (--end-to-end, --sensitive, -I 200, -X 400) (12), and the read count of each gene was normalized by the gene length (16), generating the abundance matrix of unique gene catalogue. The unique genes were compared to the NCBI NR database (blastp, e-value ≤ 1e-5) using DIAMOND v0.9.30 (17). For each sequence, the resulting alignment sequences with an e-value ≤ minimum e-value × 10 were selected. After filtering (as there may be multiple alignment results for each sequence), the multiple taxonomic information of different species was obtained. To ensure its biological significance, the Lowest Common Ancestor (LCA) algorithm was adopted for systematic classification, and the taxonomic level before the first branch was taken as the species annotation information of the sequences (18). For each taxonomic level (phylum, family, and genus), the abundance of each taxon in a sample was calculated as the sum of the abundance of each annotated gene in this taxon (19-21). Prokaryotic community profiles were constructed at the phylum, family, and genus levels for further statistical analysis.

To obtain the complete prokaryotic genomes and investigate which of the viral scaffolds are potential prophages inserted into the prokaryotic genomes, metagenomic binning was performed as follows. The (co-)assemblies generated by ourselves using metaSPAdes and clean reads were used for metagenomic binning by the metaWRAP v1.2 (22) with self-implemented MaxBin2 (23), metaBAT2 (24), and CONCOCT (25) binning modules. All original binning predictions were refined by the Bin_refinement module of metaWRAP, producing a superior bin set. The reassembly of the bin set was performed through the Reassemble_bins module of metaWRAP by mapping all the clean reads to the refined bin set, producing the final reassembled bins used for subsequent viral host prediction. The taxonomic annotation of each bin was performed using the GTDB-Tk v0.1.3 (26).

***Phylogenetic analysis of the MTS-VSs***

The proteomic tree of the high-quality viral genomes was constructed to show their phylogenetic relationship. First, the normalized tBLASTx scores (*S_G_*; 0 ≤ *S_G_* ≤ 1) between high-quality viral genomes and reference viral genomes from the Virus-Host DB database (27) and IMG/VR 3.0 database (28), were calculated using the tblastx module of the ViPTreeGen v1.1.2 (29). The *S_G_* value of pairwise genomes was calculated as previously reported (30). In detail, firstly, the high scoring segment pairs (HSPs) (31) of a query genome and a subject genome were identified using tBLASTx. For each base position of the query genome, length-normalized bit scores (i.e., HSP scores divided by HSP lengths) were assigned. Only the highest normalized score of each base position was retained. Finally, the *S_G_* value was defined as the sum of the length-normalized scores of the query genome. The reference viral genomes that show sequence similarities (*S_G_* > 0.15) to at least one of our high-quality viral genomes were then selected as the references in the proteomic tree. Here, all high-quality marine viral genomes (completeness > 90%) in the IMG/VR 3.0 database were employed to obtain the reference viral genomes. Finally, 8 MTS high-quality viral genomes homologous (*S_G_* > 0.15) to that of eukaryotic viruses were selected to build a proteomic tree of eukaryotic viruses using ViPTree online server (Host categories: Eukaryote) (29). The remaining 103 MTS high-quality viral genomes and 230 homologous reference genomes were combined, and a proteomic tree was generated by BIONJ based on the genomic distances (i.e., 1- *S_G_*) using the standalone ViPTreeGen with a threshold of 10^-3^ for the E-value. The protein tree was visualized using iTOL v6 (32). The reference high-quality viral genome sequences from IMG/VR database used in the proteomic tree are publicly available at https://doi.org/10.6084/m9.figshare.c.5703367.v6.

For the phylogenetic analysis of the viral *terL* genes, an in-house profile hidden Markov model of TerL protein was downloaded from the Pfam database (Terminase_6N, PF03237). All viral proteins from MTS-VSs and IMG/VR v3.0 database were searched against this TerL model using HMMER, hmmsearch (version 3.3.1, http://hmmer.org/) with a threshold E-value of < 1e-10. MTS viral TerL amino acid sequences were compared to those from IMG/VR v3.0 database using Diamond blastp (E-value < 10^-5^). The top-50 hits per query were extracted and dereplicated on the basis of sequence names as the reference sequences. MTS viral TerL amino acid sequences and reference sequences were merged, aligned with MAFFT (-linsi, v7.471) (33), and trimmed with trimal (-gt 0.1, v1.4.rev15) (34). The maximum-likelihood phylogenetic tree was inferred with 1,000 bootstraps using IQ-tree v1.6.12 (35) with ultrafast bootstrap and LG + R10 as suggested by model test (-m MFP, -bb 1000, -alrt 1000). Finally, the tree was visualized using iTOL v6 (32). The reference TerL amino acid sequences from IMG/VR database used in this phylogenetic tree are publicly available at https://doi.org/10.6084/m9.figshare.c.5703367.v6.

**References**

1. Wang J, Wu Y, Zhou J, Bing H, Sun H. 2016. Carbon demand drives microbial mineralization of organic phosphorus during the early stage of soil development. Biol Fertility Soils 52:825-839. doi:10.1007/s00374-016-1123-7.

2. Murphy J, Riley JP. 1962. A modified single solution method for the determination of phosphate in natural waters. Anal Chim Acta 27:31-36. doi:https://doi.org/10.1016/S0003-2670(00)88444-5.

3. Jing H, Xia X, Liu H, Zhou Z, Wu C, Nagarajan S. 2015. Anthropogenic impact on diazotrophic diversity in the mangrove rhizosphere revealed by nifH pyrosequencing. Front Microbiol 6:1172. doi:10.3389/fmicb.2015.01172.

4. Schlitzer R. 2002. Interactive analysis and visualization of geoscience data with Ocean Data View. Comput Geosci-UK 28:1211-1218. doi:https://doi.org/10.1016/S0098-3004(02)00040-7.

5. Wessel P, Luis JF, Uieda L, Scharroo R, Wobbe F, Smith WHF, Tian D. 2019. The Generic Mapping Tools Version 6. Geochem Geophys Geosyst 20:5556-5564. doi:10.1029/2019gc008515.

6. Luo M, Gieskes J, Chen L, Shi X, Chen D. 2017. Provenances, distribution, and accumulation of organic matter in the southern Mariana Trench rim and slope: Implication for carbon cycle and burial in hadal trenches. Mar Geol 386:98-106. doi:10.1016/j.margeo.2017.02.012.

7. Hiraoka S, Hirai M, Matsui Y, Makabe A, Minegishi H, Tsuda M, Juliarni, Rastelli E, Danovaro R, Corinaldesi C, Kitahashi T, Tasumi E, Nishizawa M, Takai K, Nomaki H, Nunoura T. 2020. Microbial community and geochemical analyses of trans-trench sediments for understanding the roles of hadal environments. ISME J 14:740-756. doi:10.1038/s41396-019-0564-z.

8. Maeda L, Kawahata H, Nohara M. 2002. Fluctuation of biogenic and abiogenic sedimentation on the Shatsky Rise in the western North Pacific during the late Quaternary. Mar Geol 189:197-214. doi:https://doi.org/10.1016/S0025-3227(02)00405-X.

9. Schmidt C, Sattarova VV, Katrynski L, Martínez Arbizu P. 2019. New insights from the deep: Meiofauna in the Kuril-Kamchatka Trench and adjacent abyssal plain. Prog Oceanogr 173:192-207. doi:10.1016/j.pocean.2019.02.010.

10. Wei M, Xu K. 2020. New insights into the virus-to-prokaryote ratio (VPR) in marine sediments. Front Microbiol 11:1102. doi:10.3389/fmicb.2020.01102.

11. Luo R, Liu B, Xie Y, Li Z, Huang W, Yuan J, He G, Chen Y, Pan Q, Liu Y, Tang J, Wu G, Zhang H, Shi Y, Liu Y, Yu C, Wang B, Lu Y, Han C, Cheung DW, Yiu S-M, Peng S, Xiaoqian Z, Liu G, Liao X, Li Y, Yang H, Wang J, Lam T-W, Wang J. 2012. SOAPdenovo2: an empirically improved memory-efficient short-read de novo assembler. GigaScience 1. doi:10.1186/2047-217x-1-18.

12. Langmead B, Salzberg SL. 2012. Fast gapped-read alignment with Bowtie 2. Nat Methods 9:357-359. doi:10.1038/nmeth.1923.

13. Nielsen HB, Almeida M, Juncker AS, Rasmussen S, Li J, Sunagawa S, Plichta DR, Gautier L, Pedersen AG, Le Chatelier E, Pelletier E, Bonde I, Nielsen T, Manichanh C, Arumugam M, Batto J-M, Quintanilha dos Santos MB, Blom N, Borruel N, Burgdorf KS, Boumezbeur F, Casellas F, Doré J, Dworzynski P, Guarner F, Hansen T, Hildebrand F, Kaas RS, Kennedy S, Kristiansen K, Kultima JR, Léonard P, Levenez F, Lund O, Moumen B, Le Paslier D, Pons N, Pedersen O, Prifti E, Qin J, Raes J, Sørensen S, Tap J, Tims S, Ussery DW, Yamada T, Nielsen HB, Almeida M, Juncker AS, Rasmussen S, et al. 2014. Identification and assembly of genomes and genetic elements in complex metagenomic samples without using reference genomes. Nat Biotechnol 32:822-828. doi:10.1038/nbt.2939.

14. Zhu W, Lomsadze A, Borodovsky M. 2010. Ab initio gene identification in metagenomic sequences. Nucleic Acids Res 38:e132-e132. doi:10.1093/nar/gkq275.

15. Fu L, Niu B, Zhu Z, Wu S, Li W. 2012. CD-HIT: accelerated for clustering the next-generation sequencing data. Bioinformatics 28:3150-2. doi:10.1093/bioinformatics/bts565.

16. Le Chatelier E, Nielsen T, Qin J, Prifti E, Hildebrand F, Falony G, Almeida M, Arumugam M, Batto J-M, Kennedy S, Leonard P, Li J, Burgdorf K, Grarup N, Jørgensen T, Brandslund I, Nielsen HB, Juncker AS, Bertalan M, Levenez F, Pons N, Rasmussen S, Sunagawa S, Tap J, Tims S, Zoetendal EG, Brunak S, Clément K, Doré J, Kleerebezem M, Kristiansen K, Renault P, Sicheritz-Ponten T, de Vos WM, Zucker J-D, Raes J, Hansen T, Guedon E, Delorme C, Layec S, Khaci G, van de Guchte M, Vandemeulebrouck G, Jamet A, Dervyn R, Sanchez N, Maguin E, Haimet F, Winogradski Y, Cultrone A, et al. 2013. Richness of human gut microbiome correlates with metabolic markers. Nature 500:541-546. doi:10.1038/nature12506.

17. Buchfink B, Xie C, Huson DH. 2015. Fast and sensitive protein alignment using DIAMOND. Nat Methods 12:59. doi:10.1038/nmeth.3176.

18. Huson DH, Mitra S, Ruscheweyh HJ, Weber N, Schuster SC. 2011. Integrative analysis of environmental sequences using MEGAN4. Genome Res 21:1552-60. doi:10.1101/gr.120618.111.

19. Li J, Jia H, Cai X, Zhong H, Feng Q, Sunagawa S, Arumugam M, Kultima JR, Prifti E, Nielsen T, Juncker AS, Manichanh C, Chen B, Zhang W, Levenez F, Wang J, Xu X, Xiao L, Liang S, Zhang D, Zhang Z, Chen W, Zhao H, Al-Aama JY, Edris S, Yang H, Wang J, Hansen T, Nielsen HB, Brunak S, Kristiansen K, Guarner F, Pedersen O, Doré J, Ehrlich SD, Pons N, Le Chatelier E, Batto J-M, Kennedy S, Haimet F, Winogradski Y, Pelletier E, LePaslier D, Artiguenave F, Bruls T, Weissenbach J, Turner K, Parkhill J, Antolin M, Casellas F, et al. 2014. An integrated catalog of reference genes in the human gut microbiome. Nat Biotechnol 32:834-841. doi:10.1038/nbt.2942.

20. Karlsson FH, Fåk F, Nookaew I, Tremaroli V, Fagerberg B, Petranovic D, Bäckhed F, Nielsen J. 2012. Symptomatic atherosclerosis is associated with an altered gut metagenome. Nat Commun 3:1245. doi:10.1038/ncomms2266.

21. Feng Q, Liang S, Jia H, Stadlmayr A, Tang L, Lan Z, Zhang D, Xia H, Xu X, Jie Z, Su L, Li X, Li X, Li J, Xiao L, Huber-Schönauer U, Niederseer D, Xu X, Al-Aama JY, Yang H, Wang J, Kristiansen K, Arumugam M, Tilg H, Datz C, Wang J. 2015. Gut microbiome development along the colorectal adenoma–carcinoma sequence. Nat Commun 6:6528. doi:10.1038/ncomms7528.

22. Uritskiy GV, DiRuggiero J, Taylor J. 2018. MetaWRAP-a flexible pipeline for genome-resolved metagenomic data analysis. Microbiome 6:158. doi:10.1186/s40168-018-0541-1.

23. Wu YW, Simmons BA, Singer SW. 2016. MaxBin 2.0: an automated binning algorithm to recover genomes from multiple metagenomic datasets. Bioinformatics 32:605-607. doi:10.1093/bioinformatics/btv638.

24. Kang DD, Li F, Kirton E, Thomas A, Egan R, An H, Wang Z. 2019. MetaBAT 2: an adaptive binning algorithm for robust and efficient genome reconstruction from metagenome assemblies. PeerJ 7:e7359. doi:10.7717/peerj.7359.

25. Alneberg J, Bjarnason BS, de Bruijn I, Schirmer M, Quick J, Ijaz UZ, Lahti L, Loman NJ, Andersson AF, Quince C. 2014. Binning metagenomic contigs by coverage and composition. Nat Methods 11:1144-6. doi:10.1038/nmeth.3103.

26. Chaumeil PA, Mussig AJ, Hugenholtz P, Parks DH. 2019. GTDB-Tk: a toolkit to classify genomes with the Genome Taxonomy Database. Bioinformatics 36: 1925-1927. doi:10.1093/bioinformatics/btz848.

27. Mihara T, Nishimura Y, Shimizu Y, Nishiyama H, Yoshikawa G, Uehara H, Hingamp P, Goto S, Ogata H. 2016. Linking virus genomes with host taxonomy. Viruses 8:66. doi:10.3390/v8030066.

28. Roux S, Paez-Espino D, Chen IA, Palaniappan K, Ratner A, Chu K, Reddy TBK, Nayfach S, Schulz F, Call L, Neches RY, Woyke T, Ivanova NN, Eloe-Fadrosh EA, Kyrpides NC. 2020. IMG/VR v3: an integrated ecological and evolutionary framework for interrogating genomes of uncultivated viruses. Nucleic Acids Res 49:D764-D775. doi:10.1093/nar/gkaa946.

29. Nishimura Y, Yoshida T, Kuronishi M, Uehara H, Ogata H, Goto S. 2017. ViPTree: the viral proteomic tree server. Bioinformatics 33:2379-2380. doi:10.1093/bioinformatics/btx157.

30. Bhunchoth A, Blanc-Mathieu R, Mihara T, Nishimura Y, Askora A, Phironrit N, Leksomboon C, Chatchawankanphanich O, Kawasaki T, Nakano M, Fujie M, Ogata H, Yamada T. 2016. Two asian jumbo phages, varphiRSL2 and varphiRSF1, infect Ralstonia solanacearum and show common features of varphiKZ-related phages. Virology 494:56-66. doi:10.1016/j.virol.2016.03.028.

31. Mizuno CM, Rodriguez-Valera F, Kimes NE, Ghai R. 2013. Expanding the marine virosphere using metagenomics. PLoS Genet 9:e1003987. doi:10.1371/journal.pgen.1003987.

32. Letunic I, Bork P. 2019. Interactive Tree Of Life (iTOL) v4: recent updates and new developments. Nucleic Acids Res 47:W256-W259. doi:10.1093/nar/gkz239.

33. Katoh K, Standley DM. 2013. MAFFT multiple sequence alignment software version 7: improvements in performance and usability. Mol Biol Evol 30:772-80. doi:10.1093/molbev/mst010.

34. Capella-Gutiérrez S, Silla-Martínez JM, Gabaldón T. 2009. trimAl: a tool for automated alignment trimming in large-scale phylogenetic analyses. Bioinformatics 25:1972-1973. doi:10.1093/bioinformatics/btp348.

35. Nguyen L-T, Schmidt HA, von Haeseler A, Minh BQ. 2014. IQ-TREE: A fast and rffective stochastic algorithm for estimating maximum-likelihood phylogenies. Mol Biol Evol 32:268-274. doi:10.1093/molbev/msu300.
